# Gene expression of tight junctions in foreskin is not affected by HIV pre-exposure prophylaxis

**DOI:** 10.3389/fimmu.2024.1415475

**Published:** 2024-11-06

**Authors:** Emily L. Webb, Stefan Petkov, Heejin Yun, Laura Else, Limakatso Lebina, Jennifer Serwanga, Azure-Dee A. P. Pillay, Thabiso B. Seiphetlo, Susan Mugaba, Patricia Namubiru, Geoffrey Odoch, Daniel Opoka, Andrew S. Ssemata, Pontiano Kaleebu, Saye Khoo, Neil Martinson, Julie Fox, Clive M. Gray, Carolina Herrera, Francesca Chiodi

**Affiliations:** ^1^ Medical Research Council (MRC) International Statistics and Epidemiology Group, London School of Hygiene and Tropical Medicine, London, United Kingdom; ^2^ Department of Microbiology, Tumor and Cell Biology, Karolinska Institutet, Stockholm, Sweden; ^3^ Department of Infectious Disease, Imperial College London, London, United Kingdom; ^4^ Department of Molecular and Clinical Pharmacology, University of Liverpool, Liverpool, United Kingdom; ^5^ Perinatal HIV Research Unit, University of the Witwatersrand, Johannesburg, South Africa; ^6^ Medical Research Council (MRC)/Uganda Virus Research Institute (UVRI) and London School of Hygiene and Tropical Medicine (LSHTM) Uganda Research Unit, Entebbe, Uganda; ^7^ Life Sciences & Medicine, King’s College London, London, United Kingdom; ^8^ Division of Molecular Biology and Human Genetics, Biomedical Research Institute, Stellenbosch University, Cape Town, South Africa

**Keywords:** emtricitabine tenofovir, pre-exposure prophylaxis PrEP, foreskin, transcriptomes, tight junctions, cytokines

## Abstract

**Introduction:**

Tight junctions (TJs) serve as permeability filters between the internal and external cellular environment. A large number of proteins have been identified to be localized at the TJs. Due to limitations in tissue collection, TJs in the male genital tract have been understudied.

**Methods:**

We analysed the transcriptomics of 132 TJ genes in foreskin tissue of men requesting voluntary medical male circumcision (VMMC) and enrolled in the Combined HIV Adolescent Prevention Study (CHAPS) trial conducted in South Africa and Uganda (NCT03986970). The trial evaluated the dose requirements for event-driven HIV pre-exposure prophylaxis (PrEP) with emtricitabine-tenofovir (FTC-TDF) or emtricitabine-tenofovir alafenamide (FTC-TAF) during insertive sex. A total of 144 participants were randomized to either control arm or one of 8 PrEP arms (n=16/arm), receiving oral FTC-TDF or FTC-TAF over one or two days. Following *in vivo* oral PrEP dosing and VMMC, the expression level of three important TJ proteins (CLDN-1, OCN and ZO-1) was measured *ex vivo* in foreskin tissue by Western blot. The expression of cytokine genes implicated in TJ regulation was determined. Non-parametric Kruskal-Wallis tests were used to compare TJ gene expression and protein levels by type of PrEP received, and Spearman’s correlation coefficients were calculated to assess whether TJ gene expression levels were related to cytokine gene levels or to PrEP drug concentrations and their active intracellularly phosphorylated metabolites.

**Results:**

A high level of expression in foreskin tissue was found for 118 (of 132) TJ genes analysed; this finding contributed to create a map of TJ components within the male genital tract. Importantly, PrEP regimens tested in the CHAPS trial did not affect the expression of TJ genes and the analysed proteins in the foreskin; thus, further supporting the safety of this prevention strategy against HIV-1 transmission during insertive sex. Additionally, we identified the level of several cytokines’ genes to be correlated to TJ gene expression: among them, IL-18, IL-33 and VEGF.

**Discussion:**

TJs can limit viral entry into target cells; to affect this biological function viruses can reduce the expression of TJ proteins. Our study, on the expression and regulation of TJs in the foreskin, contribute important knowledge for PrEP safety and further design of HIV-1 prophylaxis.

## 1 Introduction

Adhesion between epithelial cells is provided by desmosomes, gap junctions, adherens junctions and tight junctions (1). The latter are intercellular adhesion complexes constituting the continuous intercellular barrier between epithelial cells which serves as a permeability filter by dividing the internal and external cellular milieus, thus controlling the movement of solutes across the epithelium (2). Networks of paired, spatially organized, TJ strands form a ring surrounding the cell, which regulate the diffusion of solutes throughout the cellular sheet. Several proteins are localized at the TJs, including integral membrane proteins as well as cytosolic adaptor proteins. Transmembrane proteins include claudins (CLDNs), protein crumbs homologue 3 (CRB3), MARVEL domain proteins such as occludin (OCLN), blood vessel epicardial substance (BVES), junctional adhesion molecules (JAMS), and other immunoglobulin (Ig)-type adhesion proteins such as nectins and e-cadherin. CLDNs form strands of similar sizes that can result from the polymerization of different CLDNs. The channels established by CLDN strands regulate the paracellular ions and solutes movement between epithelial cells. Similarly, OCLN regulates the paracellular passage between cells; however, it polymerizes forming short strand fragments. Functionality of transmembrane proteins is interlinked with their interactions to cytosolic adaptors, such as zonula occludens (ZO) proteins, which connect TJs to the actin-cytoskeleton and adherens junctions [reviewed in (2)]. ZO-1 is involved in the initial steps of TJ formation between cells forming primordial junctions where they couple the assembly of transmembrane proteins to form mature TJs (3, 4). The protein composition of TJs may be dictated by the functional properties of the different epithelial cell types in tissues including cell polarity, signaling and vesicle trafficking [reviewed in (5)]. The number of proteins considered part of the TJs family has been progressively growing during the recent years; approximately 130 genes are currently listed in the gene ontology term “cell-cell junction assembly” (https://amigo.geneontology.org/amigo/term/GO:0007043). Multiple functions are included under this gene ontology class allowing gene classification under different major subfamilies (**Supplementary Table 1**). The formation of TJs involves a high degree of functional redundancy as shown in each family; for example, CLDN1 and OCLN, two transmembrane proteins, are part of the “bicellular tight junction assembly” class. CLDN1 and OCLN interact with the cytosolic adaptor ZO-1, encoded by gene TJP1 which belongs to the gene ontology family of “cell-cell junction assembly”.

The abundance of TJ proteins in tissue is regulated by different cytokines. As examples, CLDN1 expression is down-regulated by TGFβ1 (6) and IL-33 (7), whereas TNF-α increases the expression of CLDN 1, 4 and 7 in tubular cells (8). Inflammatory conditions may also be a dramatic driving force for changes in the expression of TJ proteins. In this respect, it was shown that local cytokine production in the labial salivary gland was impaired in Sjögren’s syndrome patients, with this alteration affecting the TJs’ integrity of epithelial cells, a condition which could be mimicked *in vitro* by TNF-α and IFN-γ (9).

In view of their role as physical barriers, TJs can block viral entry into host cells. Viruses, however, have been shown to impair epithelial barriers for the purpose of increasing infection rate of target cells [reviewed in (10)]; this can be achieved by affecting the expression of TJ proteins and function, including permeability (11). The list of viruses able to promote infection of target cells by affecting TJs is comprehensive and includes retroviruses (HIV), flaviviruses (HCV, ZIKV, DENV), rabies virus and respiratory viruses (IAV, RSV, SARS-CoV, rotavirus) (12); these families of viruses have developed their own unique molecular strategies to utilize TJs for the purpose of infecting target tissues. HIV-1 was shown to induce down-regulation of TJ expression, particularly CLDN 2, OCLN and ZO-1 at the vaginal barrier (11–13) likely leading to increased permeability of the tissue to HIV-1 (12); down-regulation of TJ expression induced by viruses can also alter the epithelial TJ barrier role as sensors of host innate immune system (14).

The difficulty of obtaining tissue specimens hampers the possibility of building a complete profile of TJs in the human male genital tract, as well as the characterization of factors that can modulate their expression. The results obtained from randomized controlled trials conducted in Sub Saharan Africa demonstrated that voluntary medical male circumcision (VMMC) provided over 50% protection from HIV infection (15–17). The mechanism for the protective effect of VMMC is not fully clarified; however, as the foreskin carries high densities of HIV target cells (CD4+ T cells and Langerhans cells), the most likely mechanism is removal of tissue containing HIV target cells (18–20). VMMC has represented an important source of foreskin tissue useful to dissect physiological and pathological events in the male genital tract. A decreased expression of CLDN-1 was detected in the foreskin of asymptomatic HSV-2 seropositive individuals suggesting a fragile epithelial barrier in the genital tract which could increase the risk of contracting HIV-1 infection (21). A study on the foreskin of men who have sex with men or transgender women revealed subclinical changes in the inner foreskin, symptomatic of an inflammatory state with the potential of modifying epidermal barriers and availability of target cells for HIV-1 infection (22). Furthermore, ex vivo pre-clinical studies with human tissues (23–28) and clinical trials (29–32) have shown that event-driven oral and topical PrEP induce changes at the proteomic and transcriptomic levels in the female and male genital tracts as well as in the colorectum. However, no studies have assessed in parallel the impact of PrEP at the proteomic and transcriptomic levels in foreskin tissue and specifically the potential effect on epithelial integrity.

A recent open label randomized controlled trial (NCT03986970) conducted by the CHAPS consortium in South Africa and Uganda evaluated the dose for event-driven HIV PrEP for insertive sex (33). The trial included HIV-1 negative males requesting VMMC who were randomized to a control arm or to receive emtricitabine-tenofovir (FTC-TDF) or emtricitabine-tenofovir alafenamide (FTC-TAF) over one or two days. The primary outcome was to determine ex vivo protection against HIV-1 challenge of foreskin tissue samples (obtained at VMMC) (33). The results of the trial showed that all regimens tested with either FTC-TDF or FTC-TAF given prior to VMMC provided protection against ex vivo HIV-1 challenge. Ex vivo analysis of foreskin tissue from the CHAPS trial participants revealed that *in vivo* short-course of event-driven oral PrEP in men induced changes at the transcriptomic level, including modulation of genes involved in inflammation, mitochondrial function and cell proliferation (34).

As the number of individuals receiving HIV PrEP continues to grow world-wide, it is of high interest to study whether PrEP induces changes in the biology of the male genital tract which could be beneficial, or detrimental, for HIV protection. Hence, we further analyzed the data collected from the CHAPS trial focusing on the genes coding for TJ proteins in foreskin. The study provides the expression profile of the foreskin TJs and cytokine genes influencing their expression in this tissue.

## 2 Materials and methods

### 2.1 Study population and sampling

The CHAPS trial recruited HIV negative males aged 13-24 years from VMMC clinics at Chris Hani Baragwanath Academic Hospital, Soweto, South Africa and Entebbe General Hospital, Entebbe, Uganda. Participants were randomized to either a control arm (receiving no PrEP) or PrEP trial arms where they received orally either FTC-TDF or FTC-TAF over two days or one day, before undergoing VMMC. Following *in vivo* dosing, or not for the control arm, samples including foreskin tissue, peripheral blood mononuclear cells (PBMCs) and plasma were collected from all individuals at time of VMMC, for ex vivo analyses. Resected inner and outer foreskin tissues were processed in the laboratory and cut into explants of different sizes specific for each ex vivo assay. Comparative analysis of outer and inner foreskins was not in the scope of this trial, and for each analysis an explant of outer was mixed with a piece of inner tissue. Full details of the CHAPS trial design and results for primary and key secondary outcomes have been described previously (33). At the circumcision visit, all participants provided midstream urine for *Chlamydia trachomatis* and *Neisseria gonorrhea* testing via nucleic acid amplification testing (NAAT) prior to surgery.

### 2.2 Transcriptomic analysis

Details on preparation of resected foreskin and RNA sequencing of this tissue, including RNA-seq data processing and analysis were published (34). Briefly, total RNA was isolated from homogenized foreskin tissue samples (RNeasy kit, Qiagen, Hilden, Germany) and cDNA libraries prepared for Illumina sequencing with an Illumina Novaseq 6000 S4 flowcell (Illumina, San Diego, CA, USA). Reads were aligned to the Ensembl GRCh38 reference genome using STAR (v2.6.1d). Counts for each gene were obtained using featureCounts (v1.5.1). The transcriptome data presented in the study are deposited in the GenBank Data Libraries repository, accession number PRJNA884284.

### 2.3 Western blot analysis

For each participant, an inner and an outer foreskin explant of approximately 10 mg each were cut, pooled, and dry frozen at -80°C until analysis. Explants were lysed with a solution of Tris-HCl (pH 7.4) and 1x complete protease inhibitor cocktail (MilliporeSigma, Darmstadt, Germany) in Lysing Matrix A (MP Bio-medicals, Santa Ana, CA, USA), and homogenized with a FastPrep^®^ Ribolyser FP120 (Thermo Scientific Savant™, Waltham, MA, USA). Lysed tissues were spun down and supernatants transferred to Vivaspin^®^ 500 tubes (Sartorius, Göttingen, Germany) to concentrate the lysates. Recovered protein concentrates were quantified with Pierce TM BCA Protein Assay kit (Thermo Scientific). Ten µg of protein/well were loaded in a 9% SDS-polyacrylamide gel and transferred to nitrocellulose membranes (Bio-Rad, Hercules, CA, USA). The blots were blocked in PBS-5% skimmed milk overnight and then probed with rabbit anti-OCLN Ab (1:500; ab167161 Abcam, Cambridge, UK), mouse anti-CLDN-1 Ab (1:125; 37-4900 Invitrogen, Waltham, MA, USA) and rabbit anti-ZO1 Ab (1:5,000; ab2272 Sigma-Aldrich, Burlington, MA, USA). Membranes were then incubated with secondary-horseradish peroxidase (HRP) antibodies (HRP anti-rabbit IgG: 1:2,000; NA 943 and HRP anti-mouse IgG: 1:2,000; NA 931 GE Healthcare, Chicago, IL, USA). Proteins bands were visualized with ECL ClarityTM Western ECL Substrate (Bio-Rad). Normalization of TJ protein signals was conducted via detection of β-actin levels (HRP mouse anti-β-actin Ab, 1:10,000; ab49900 Abcam). Western blot images were captured by Azure c300 Imaging System (Azure biosystem, Dublin, CA, USA) and analyzed with Image Studio Lite software Ver 5.2 (LI-COR Bioscience, Lincoln, NE, USA).

### 2.4 Pharmacokinetics and pharmacodynamics analysis

Concentrations of TFV, FTC, the pro-drug TAF, and the active phosphorylated intracellular metabolites, tenofovir-diphosphate (TFV-DP) and emtricitabine-triphosphate (FTC-TP), were determined in foreskin tissue as described previously (35). Briefly, for each participant, inner and outer foreskin explants of 10 mg each were cut and frozen at -80°C in ice-cold chelating solution, methanol and 20 mM EDTA-EGTA (70:30 V/V). Explants were homogenized and analyte quantification was performed using a SCIEX 4500 or 5500 triple quadrupole mass spectrometer (AB Sciex UK Limited; Warrington, UK). Drug and metabolite concentrations in foreskin were quantified using a ng/sample or pmol/sample calibration curve and values normalized to ng/g or pmol/g of tissue.

Ex vivo susceptibility to HIV-1 infection was evaluated as described previously (33). Briefly, foreskin tissues resected by VMMC from each trial participant, were cut into explants and transferred to 96-well U-bottom plates in a non-polarized system. Explants were challenged ex vivo with HIV-1_BaL_ for 20 h in the absence of drug. Infectivity was measured during 15 days of culture by analysis of p24 concentration in culture supernatants (Innotest HIV antigen ELISA, Fujirebio Europe, Ghent, Belgium).

### 2.5 Study approval

Ethical clearance to conduct the trial was obtained from the South African Health Products Regulatory Authority (20181004); the Uganda Virus Research Institute Research Ethics Committee (GC/127/18/12/680); Uganda National Council of Science and Technology (HS 2534); Uganda National Drug Authority (618/NDA/DPS/09/2019) and the London School of Hygiene and Tropical Medicine Research Ethics Committee (Ref:17403). Informed written consent was collected from all participants. The Swedish Ethics Review Authority approved the laboratory studies of the collected specimens at the Karolinska Institutet (2020-00941).

### 2.6 Data analysis

Participant characteristics were summarized with frequencies for categorical variables and means and standard deviations for continuous variables. Characteristics of participants were compared between South Africa and Uganda using Wilcoxon ranksum test to compare age (as non-normally distributed), t-test to compare BMI, and chi-squared tests to compare trial arm. The distributions of TJ gene expression levels, foreskin genes encoding cytokines and TJ proteins were visualized and summarized as medians with interquartile ranges. Since distributions of these measures were either skewed or bi-modal, and could not be normalized through transformation, non-parametric statistical methods were used for all analyses. Spearman’s correlation coefficients and corresponding p-values were calculated to determine associations between (1) each pair of TJ genes, (2) each pair of foreskin genes encoding cytokines, and (3) each possible pair of TJ genes and foreskin genes encoding cytokines. Since expression levels of many of the TJ genes were strongly positively correlated, principal component analysis (PCA) of TJ gene expression levels was conducted as a data reduction technique to generate a new variable (the first principal component) comprising a linear combination of the TJ gene expression levels which captured a high degree of their variability; a high value for this variable represents high expression levels for many of the TJ genes, while a low value for this variable represent low expression levels for many of the TJ genes.

The effect of oral PrEP (trial arm) was examined by comparing gene expression levels (and the first principal component representing their combination) and genes encoding cytokines between trial arms (FTC-TDF versus FTC-TAF versus no PrEP) using the Kruskal-Wallis test. These analyses were repeated, separately for each country (South Africa and Uganda). Spearman’s correlation coefficients and corresponding p-values were calculated to assess the relationship of TJ genes and genes encoding cytokines with foreskin drug levels. Levels of TJ proteins measured by western blot were compared between trial arms (FTC-TDF versus FTC-TAF versus no PrEP) using Kruskal-Wallis tests. All analyses were adjusted for multiple testing using the false discovery rate (FDR): FDR-adjusted p-values are reported throughout unless otherwise indicated.

## 3 Results

Of the 144 participants enrolled in CHAPS, foreskin TJs and cytokine gene expression as well as TJ proteins abundance data were available from 139, comprising 68 of the 72 participants enrolled in South Africa, and 71 of the 72 participants enrolled in Uganda. Characteristics of participants with data available for this analysis are summarized in **Table 1**. Overall, the median age of participants was 19 years (interquartile range 16-21 years) and the distributions of age and other characteristics were comparable across the two countries. A minority of individuals included in the trial (6 out of 139; 2 individuals from South Africa and 4 from Uganda) tested positive for *Chlamydia trachomatis* and all individuals were negative for *Neisseria gonorrhea* (**Table 1**); these results were not considered in the analyses.

**Table 1:** Characteristics of CHAPS participants included in the analysis, overall and by study site.

Characteristic	All participants N=139	South Africa N=68	Uganda N=71	p-value^1^
Sex, male	139 (100%)	68 (100%)	71 (100%)	1.00
Age in years, median (interquartile range) [range]	19 (16-21) [13-24]	20 (16-22) [13-24]	19 (16-21) [13-23]	0.51
BMI, mean (SD)	20.7 (2.9)	20.8 (3.6)	20.6 (2.0)	0.68
Chlamydia positive	6 (4%)	2 (3%)	4 (6%)	0.45
Trial arm				1.00
Control	16 (12%)	8 (12%)	8 (11%)	
FTC-TDF, 2 tablets, 5 hours	16 (12%)	8 (12%)	8 (11%)	
FTC-TDF, 2 + 1 tablets, 5 hours	16 (12%)	8 (12%)	8 (11%)	
FTC-TDF, 2 tablets, 21 hours	16 (12%)	8 (12%)	8 (11%)	
FTC-TDF, 2 + 1 tablets, 21 hours	15 (11%)	7 (10%)	8 (11%)	
FTC-TAF, 2 tablets, 5 hours	14 (10%)	7 (10%)	7 (10%)	
FTC-TAF, 2 + 1 tablets, 5 hours	15 (11%)	7 (10%)	8 (11%)	
FTC-TAF, 2 tablets, 21 hours	16 (12%)	8 (12%)	8 (11%)	
FTC-TAF, 2 + 1 tablets, 21 hours	15 (11%)	7 (10%)	8 (11%)	

^1^P-value for difference in distribution of characteristics between South Africa and Uganda participants: Wilcoxon rank-sum test used to compare age (as non-normally distributed), t-test used to compare BMI, chi-squared test used to compare sex, chlamydia result and trial arm.

**Figure 1:**
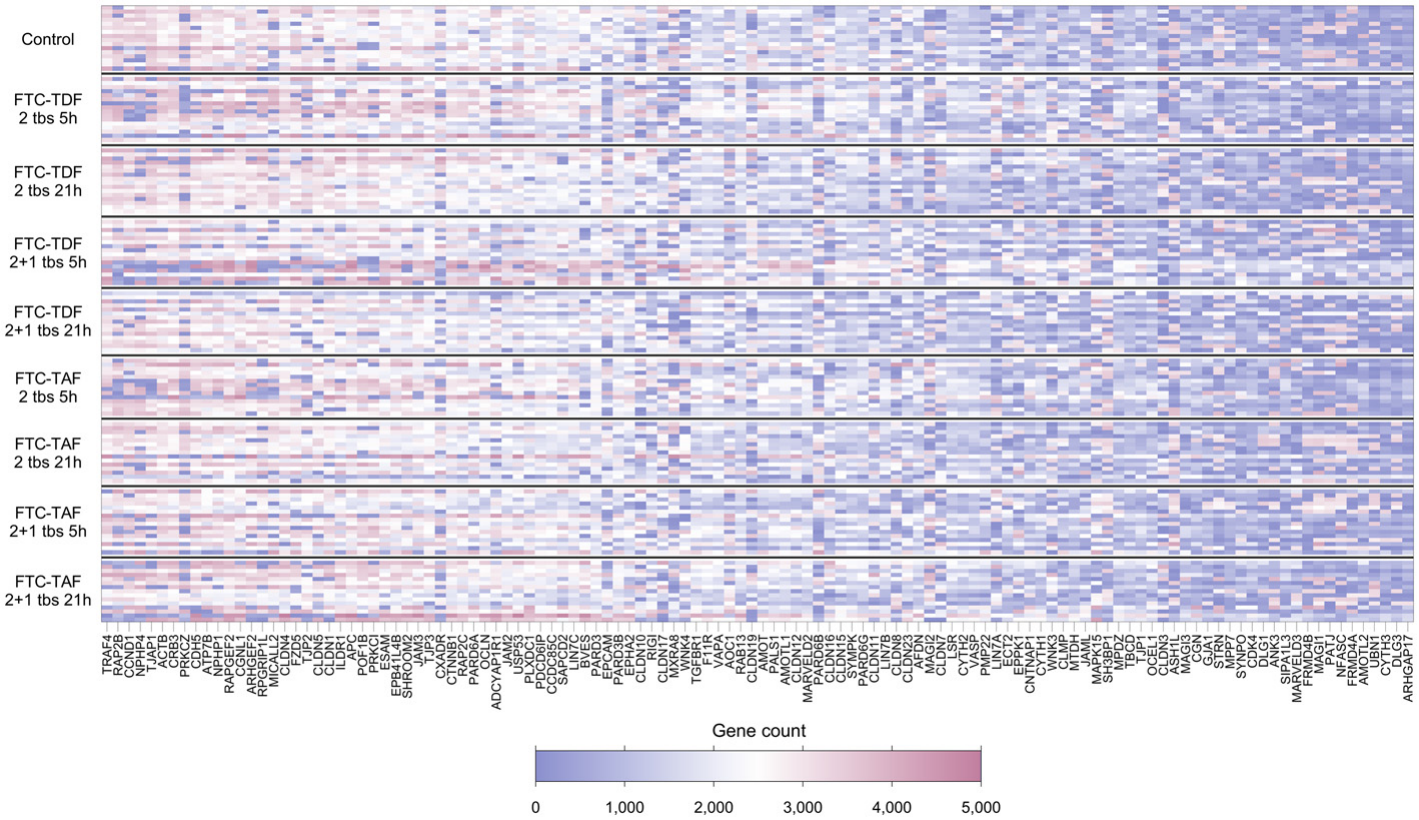
Heatmap showing expression levels (gene counts) of 118 TJ genes for 139 individuals included in the CHAPS study. Each row represents one individual, and each column represents one gene. Individuals (rows) are grouped according to their CHAPS trial arm. Genes (columns) are ordered by median gene expression level (high to low). Darker red values indicate higher gene counts while darker blue values indicate low gene counts, as shown in the legend.

Of the 132 TJ genes evaluated, 14 were excluded from further analyses as they were expressed in fewer than 70% of participants overall (**Supplementary Table 2**). **Figure 1** and **Supplementary Figure 1** show the expression levels for each participant for the remaining 118 genes, with participants ordered by trial arm and country, respectively. Among all participants combined, the 10 most highly expressed genes were *TRAF4, RAP2B, CCND1*, *NPHP4*, *TJAP1, ACTB, CRB3, PRKCZ, CDH5* and *ATP7B*; the function of proteins coded by these genes is shown in **Table 2**.

**Table 2:** Function of 10 most highly expressed tight junctions genes in foreskin samples from CHAPS participants.

Gene Name	Protein	Function of protein^1^
*TRAF4*	TNF Receptor Associated Factor 4	Adapter protein with E3 ligase activity involved in cell proliferation, migration, differentiation, DNA repair, platelet activation or apoptosis
*RAP2B*	Ras-Related Protein 2B	May play a role in cytoskeletal rearrangements and regulate cell spreading
*CCND1*	Cyclin D1	Member of highly conserved cyclin family; ubiquitously expressed in all tissues
*NPHP4*	Nephrocystin 4	Involved in the organization of apical junctions
*TJAP1*	Tight Junction Associated Protein 1	Tight junction-associated protein. Localizes to the Golgi and may function in vesicle trafficking
*ACTB*	Actin beta	Major constituent of the cell contractile apparatus
*CRB3*	Crumbs Cell Polarity Complex Component 3	Involved in the establishment of cell polarity in mammalian epithelial cells
*PRKCZ*	Protein Kinase C Zeta	Calcium- and diacylglycerol-independent serine/threonine-protein kinase. Involved in establishment of cell polarity of astrocytes
*CDH5*	Cadherin 5	May play an important role in endothelial cell biology through organization of the intercellular junctions
*ATP7B*	ATPase Copper Transporting Beta	Copper ion transmembrane transporter involved in the export of copper out of the cells

^1^UniProtKB/Swiss-Prot Summary for Genes.

Expression levels were strongly positively correlated between genes; of the 6,903 gene-gene pairs, 3,323 (48%) showed significant pairwise correlation after adjustment for multiple testing using FDR (all FDR-adjusted p-values <0.05; **Supplementary Figure 2**). In PCA analysis, the first component captured 31% of the variability in expression levels for all 118 TJ genes.

**Figure 2:**
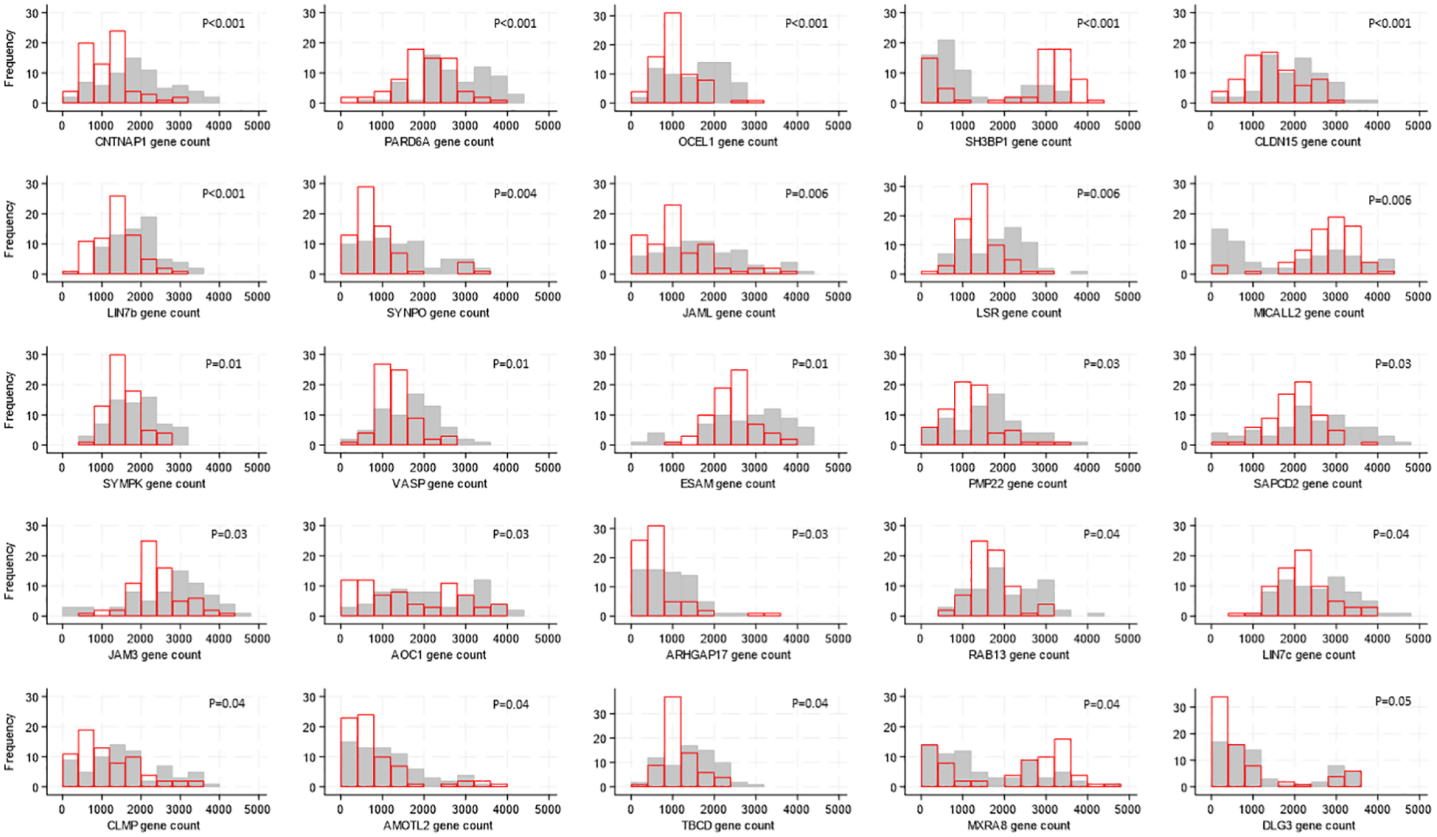
Distributions of tight junction gene expression levels by country, shown for the 25 genes whose expression levels differed by country. Gene counts are indicated by the values on the x-axis, categorized into groups of width 500, and the number of participants falling into each gene count group is shown on the y-axis. Grey bars show distribution of expression levels from South African participants, red bars show distribution of expression levels from Ugandan participants. P-values for differences in distribution of gene expression levels between countries were generated by Kruskal-Wallis tests and adjusted for multiple testing using the false discovery rate approach are indicated on each graph.

There were differences in gene expression levels between Ugandan and South African participants, with 25 of the 118 genes showing evidence of a difference (after FDR adjustment). For 22 of these 25 genes, expression was higher in samples from South African participants; the median expression ranged from 1.2 to 2-fold higher with corresponding p-values 0.04 to <0.0001 (**Figure 2**) whereas the expression of *SH3BP1* (p<0.001), *MICALL2* (p=0.006) and *MSRA8* (p=0.04) was higher in individuals from Uganda.

When examining the effect of oral PrEP through comparing gene expression levels by CHAPS trial arm, there was no impact of oral PrEP on foreskin TJ gene expression, either when comparing expression levels for each gene individually between trial arms (smallest FDR-adjusted p-value 0.14; **Supplementary Table 3**), or when comparing the score for the first principal component between trial arms (p=0.71; **Supplementary Figure 3**). This was also the case when participants from each country were analyzed separately (**Supplementary Tables 4**, **5**).

**Figure 3:**
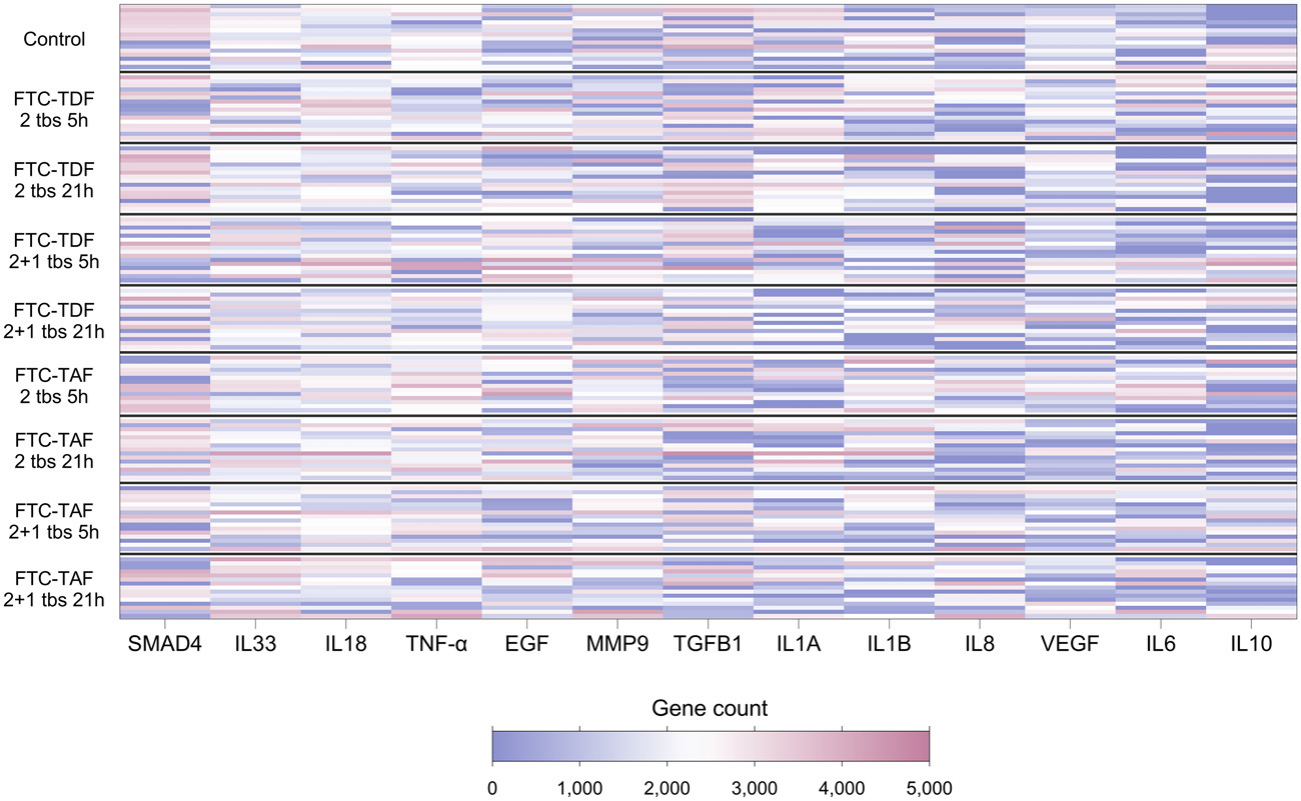
Heatmap showing expression levels of foreskin genes encoding cytokines, for 139 individuals included in the CHAPS study. Each row represents one individual, and each column represents one gene. Individuals (rows) are grouped according to their CHAPS trial arm. Genes encoding cytokines (columns) are ordered by median gene count (high to low). Darker red values indicate higher gene counts while darker blue values indicate lower gene counts, as shown in the legend.

**Figure 4:**
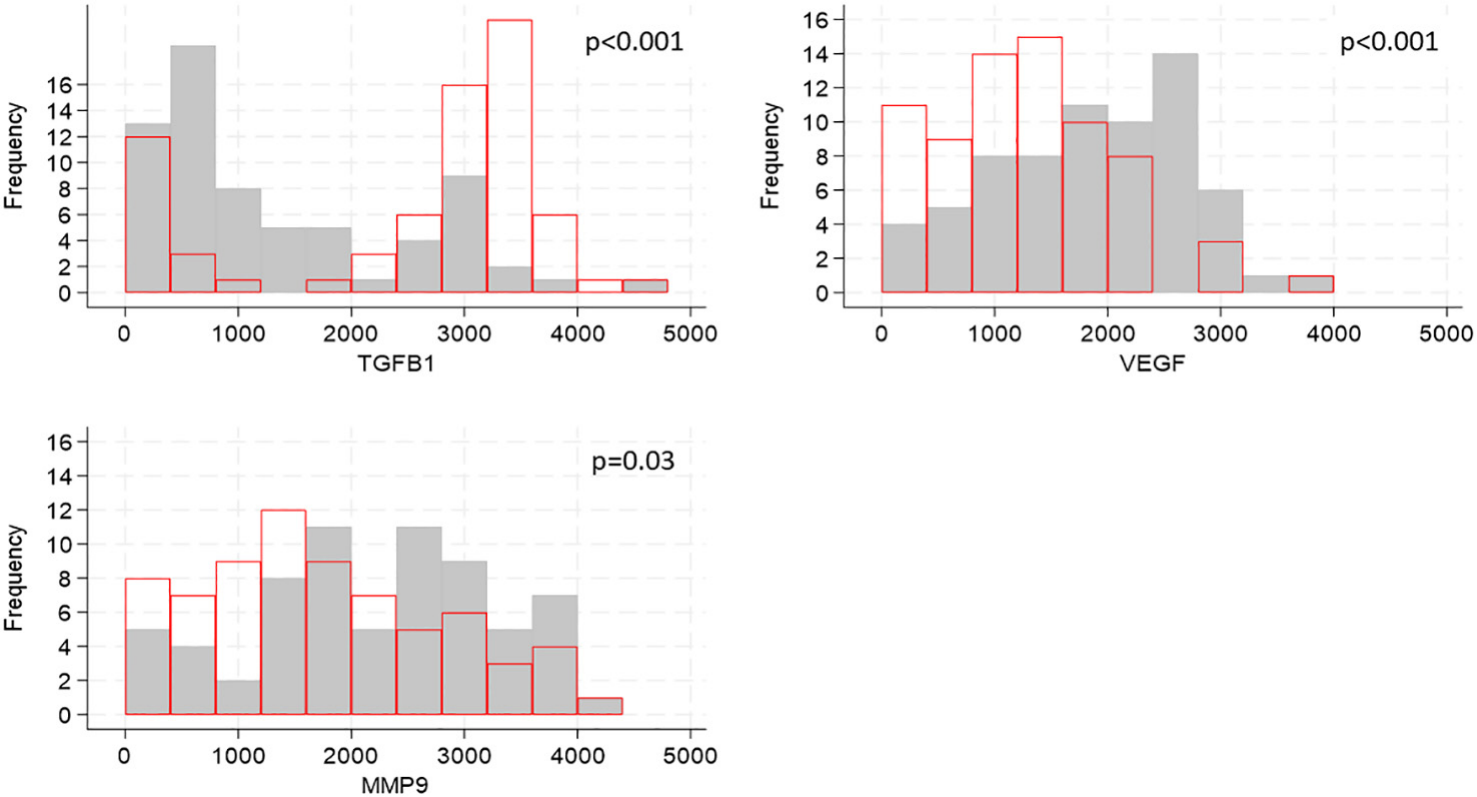
Distributions of cytokine gene expression by country, shown for genes whose distributions differed by country. Cytokine gene counts are indicated by the values on the x-axis, categorized into groups of width 400, and the number of participants falling into each group is shown on the y-axis. Grey bars show distribution of cytokine gene expression from South African participants, red bars show distribution of cytokine concentrations from Ugandan participants. P-values for differences in distributions between the two countries were generated by Kruskal-Walis tests and adjusted for multiple testing using the false discovery rate approach are indicated on each graph.

Cytokines are known to influence and regulate the expression of TJ proteins (6–8); utilizing our transcriptomic data we evaluated whether the gene expression of cytokines known to affect TJ proteins correlated with the expression of TJ genes. Of the 21 genes encoding cytokines and evaluated in foreskin, eight were detectable in less than 70% of participants and were not analyzed further (**Supplementary Table 6**). Individuals’ levels for the remaining 13 are shown in **Figure 3**. The distribution of three genes differed between South African and Ugandan participants: *TGFB1* was significantly higher in Ugandan participants (p<0.001), while *VEGF* (p<0.001) and *MMP9* (p=0.03) were significantly increased in South African participants (**Figure 4**). There was no effect of oral PrEP on the distribution of these outcomes, either overall or when countries were analyzed separately (**Supplementary Table 7**).

**Figure 5:**
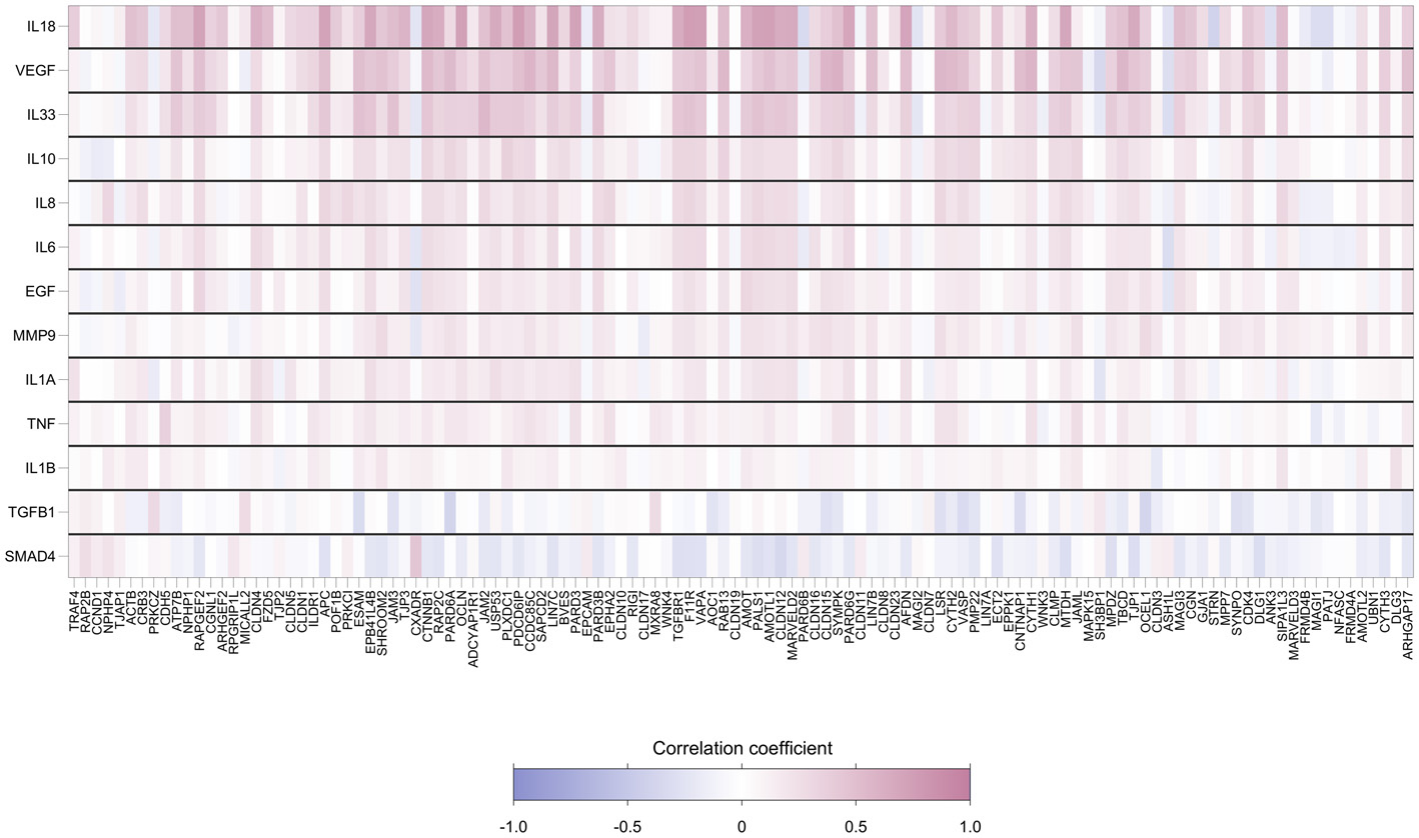
Heatmap showing pairwise correlation coefficients between expression levels of 118 foreskin tight junctions and 13 cytokine genes. Each row represents a gene encoding cytokine, and each column represents a TJ gene. Spearman’s correlation coefficient was calculated for each row-column pair. Darker red values indicate higher positive correlation while darker blue values indicate higher negative correlation, as shown in the legend.

Foreskin genes encoding cytokines were strongly positively correlated with TJ gene expression levels. Of the 1534 gene-cytokine pairs, 481 (31%) showed significant pairwise correlation (**Figure 5**). The cytokine genes most strongly correlated with levels of TJ gene expression were IL-18, VEGF and IL-33. **Supplementary Figure 4** displays the relationship of these cytokine gene counts with the score for the first principal component (which captures a large degree of the variability in expression levels for genes analyzed); correlation coefficients were 0.71, 0.56 and 0.45, respectively (all p<0.001).

We found no evidence that TJs and cytokine gene expression levels were associated with the concentrations of TFV-DP and FTC-TP measured in foreskin, either overall or in separate country-specific analyses (**Supplementary Table 8**). No evidence of correlation was found between p24 concentrations measured in foreskin tissue challenged ex vivo with HIV-1 and either TJs or cytokine gene levels (**Supplementary Table 9**). The pharmacodynamic and pharmacokinetic profiles of these drugs in foreskin have been extensively presented (33).

We then analyzed the level of expression for three TJ proteins, CLDN-1, OCLN and ZO-1, via western blot. Samples from 138 participants were available; one participant from South Africa included in the transcriptomic analysis did not have a sample available for proteomic analyses. When examining the effect of oral PrEP regimens tested in the CHAPS trial on these proteins, no differences between treatment arms were detected (**Supplementary Table 10**, **Supplementary Figure 5**).

## 4 Discussion

Our study is the first to report the complete expression profile of TJ genes in the foreskin tissue of individuals receiving oral PrEP (both TDF-FTC and TAF-FTC) in two different sub-Saharan countries, South Africa and Uganda. The results showed that the expression of TJ genes is not altered in subjects receiving short-term PrEP and that there was no correlation of expressed TJ genes with the concentrations of TFV-DP and FTC-TP measured in foreskin of these subjects nor with the ex vivo HIV-1_BaL_ infectivity levels (33).

Our consortium conducted a clinical trial comparing efficacy of FTC-TDF versus FTC-TAF dosing prior to VMMC on HIV-1_BaL_ infection of foreskin using an ex vivo challenge model. This trial simulated the relative protection that could be attained for insertive sex by pre- and post-exposure regimens. Foreskin tissue was utilized to determine dosing schedules and duration of protection against ex vivo challenge (35); as well as concentrations of drug and active metabolites in foreskin tissue. The levels of drug metabolites TFV-DP and FTC-TP in foreskin were comparable to exposures previously reported in cervical and vaginal tissues (36, 37). It is of interest that, in men included in the CHAPS trial, timing and dose of F/TDF and F/TAF had no significant impact on CD4+CCR5+ cell numbers in foreskins and on CCR5 expression levels on CD4+ cells compared to the control arm, as shown by immunohistochemistry imaging (38); these results suggested that PrEP does not alter the immunological microenvironment of the foreskin and that PrEP induced protection from HIV infection is not the result of a diminished expression of HIV co-receptor CCR5 on CD4+ cells (38).

In our study, both FTC-TDF and FTC-TAF were well tolerated and highly effective against ex vivo challenge of foreskin tissue with a clade B R5-tropic laboratory adapted isolate, HIV-1_BaL_ (33). It is relevant that in spite of the measurable levels of FTC-TDF and FTC-TAF in the foreskin tissue and the protection that these drug levels provided to HIV-1_BaL_ ex vivo challenge, the expression of foreskin TJ genes studied by us did not change significantly when the control group was compared to groups receiving either FTC-TDF or FTC-TAF. That TJ expression remains unchanged in our study in individuals receiving PrEP is an encouraging information as a large number of individuals receive PrEP world-wide. The results obtained from the short time PrEP administration tested in the CHAPS trial are corroborated by the results presented in a previous study by Hladik et al. (29) where the effect of tenofovir 1% gel on rectal mucosa was studied in individuals treated with once-daily applications for seven consecutive days. In this study, **Supplementary Materials** listed genes which were up- or down regulated in biopsies obtained at day 7 of treatment in relation to biopsies obtained at enrolment. We assessed whether any of the 118 genes included in the gene ontology term “cell-cell junction assembly” studied in the present work were dysregulated in the supplementary results presented by Hladik and co-authors (29). Interestingly, only one gene *MARVELD3* (involved in bicellular TJ assembly) was down-regulated after 7-days of treatment (29). Deletion of CLDN-1, an important TJs component, was demonstrated to be highly lethal in mice after birth because of defects in epidermal barrier function (39). We have previously performed immunohistochemistry analysis of CLDN-1 protein abundance in the foreskin tissues collected during CHAPS trial (38); the results showed that the geometric mean of percentage CLDN-1 levels was 34% higher in foreskin tissue from participants receiving PrEP, compared with the control arm, a result which was no longer significant, after allowing for multiple comparisons. In the present study, measurement of CLDN-1, OCN and ZO-1 by western blot revealed that the abundance level in foreskin tissue from the PrEP treated individuals did not differ from the control group. Considering the different body compartments studied by us and Hladik and co-authors (29), the different length of treatment and the multiple methods used for analyses we can anticipate that PrEP has a minimal impact, if any, on the expression of TJ genes.

The expression of all 118 TJ genes found in over 70% of individuals included in the present study was in general high, with read counts >500. Among the most highly expressed (>2600 copies), genes with a broad range of functions could be found; the mapping of these expressed genes to the foreskin may be useful in the context of mechanism of diseases and therapeutical approaches. It is interesting that the expression of 25 genes coding for TJ proteins differed between participants in South Africa and Uganda. Whether this difference in the expression of a group of genes reflects a specialized biological function remains to be studied. One hypothesis is that genetically determined differences, as previously reported by the African Genome Variation Project (40), or diet, could be at the basis for these differences; in this context, it was reported that the intestinal TJ barrier could be regulated by dietary factors in connection to different inflammatory disorders [reviewed in (41)]. There are 9654 genes present in the human gene database (https://www.genecards.org) which are associated to HIV; among those the expression of TJ genes *CNTNAP1, PARD6A, LIN7B, SYNPO, LSR, SYMPK, JAM3, AOC1, RAB13, LIN7C, AMOTL2, TBCD*, as detected in our study, was found to be higher in individuals from South Africa. Further studies could reveal if the increased expression of the 12 TJ genes associated with HIV in South African males render them less susceptible to HIV infection.

We found that the expression levels of the majority of TJ genes were strongly positively correlated. Correlations in gene expression can be used to deduce functional and regulatory relationships between genes; the optimal setting to study gene correlations is to use single cell transcriptomics (42). In our study, we used the whole foreskin tissue to study gene expression; considering that the studied genes all belong to the gene ontology TJs it is likely that the highly positive correlations between TJ genes further strengthen the concept that the selected genes represent a cluster which components together carry out complex biological functions.

It is of interest that foreskin genes encoding cytokines were strongly correlated with TJ gene expression levels. That expression of TJ proteins is influenced by cytokines has been reported previously in several contexts (6–9, 43, 44). In our analyses, IL-18, VEGF and IL-33 were the cytokine genes which most strongly correlated with levels of TJ gene expression. Dysregulated expression of these cytokines can be linked to several types of disorders in humans: induction of VEGF has been reported to be induced by several viruses associated with cancer (45); IL-18 has been linked to several inflammatory diseases including HIV-1 (46); IL-33 has been linked to HIV-associated neurological disorders (47). HIV-1 has been shown to induce down-regulation of TJs to facilitate infection of target cells (11–13).

Our study presents with limitations which should be taken in consideration in follow-up investigations. The function of TJ genes has been previously studied in detail in epithelial cells whereas the complete picture of TJ expression and function in other cells, including immune cells, remains incomplete. The expression of TJ genes in the present study was obtained for the whole foreskin tissue and individual cellular components were not separated. The foreskin tissue has, in fact, been shown to be rich in epithelial cells and Langerhans’ cells which are target cells for HIV-1 infection (48); in addition, dendritic cells, macrophages and T cells are also present in foreskin although they are predominantly dermal. Furthermore, a separate analysis for inner and outer foreskins was not performed. Considering the heterogeneity of HIV-target cells between inner and outer tissues (49, 50), further studies will be essential to determine any tissue-specific modulation of TJs by cytokines/chemokines and potential impact of PrEP regimes. Another important limitation in the present study is that PrEP administration in the CHAPS trial was limited to a maximum of two doses administered with a maximum interval of 24 hours; it is important for these studies to be repeated in individuals who have received PrEP for a prolonged period of time. The opportunity to conduct novel studies on the relevance of genital tissue barriers for protection from HIV or other sexually transmitted infections could be provided by trials focused on long-acting PrEP drugs. A phase 3 trial enrolling young women and adolescent girls in Uganda and South Africa (51) demonstrated that twice-yearly administration of subcutaneous Lenacapavir, a long-acting agent approved by the Food and Drug Administration (FDA) for HIV treatment, prevented HIV infection. The incidence of laboratory-diagnosed, sexually transmitted infections *C. trachomatis*, *N. gonorrhoeae*, or *T. vaginalis* infections was however high in the Lenacapavir group and comparable to groups of women receiving F/TAF or F/TDF in the same trial (51).

The mRNA gene expression has been reported to highly correlate (90%) with protein level (52); our study is mostly based on mRNA gene expression, and it would be interesting to study whether any difference can be found when methods aimed at determining protein levels in tissue will be applied to biological specimens from individuals receiving PrEP. Additional studies should verify the biological role of individual TJ proteins in the foreskin tissue. Another important aspect of the CHAPS trial is the focus on young males between the ages of 13 and 24-years old. Testosterone has been shown to modulate the expression level of TJs in the male genital tract. Expression of ZO-1 and CLDN-1 has been shown to be upregulated by testosterone (53, 54). The immune system is also affected by hormonal changes during adolescence (55), which could impact the formation of TJ in foreskin tissue. In this study we did not have statical power to identify potential changes in TJ expression levels and the potential impact of PrEP, between different age groups within the range of participants recruited.

The major findings of this study are: i) the expression map of TJ components in the foreskin tissue; ii) PrEP does not affect the expression of TJ genes and proteins in the foreskin; iii) identification of cytokines whose gene expression is linked to TJ gene expression.

## Data Availability

The datasets presented in this study can be found in online repositories. The names of the repository/repositories and accession number(s) can be found in the article/**Supplementary Material**.
