# Supplementary material for: Gene expression of tight junctions in foreskin is not affected by HIV pre-exposure prophylaxis
Source: Front Immunol. 2024 Nov 6;15:1415475. doi: 10.3389/fimmu.2024.1415475 (PMC11576434; doi:10.3389/fimmu.2024.1415475)

Supplementary Table 1. Description of major TJ gene subfamilies

**GENE ONTOLOGY CLASS**: FUNCTION*	GENES**
**Bicellular tight junction assembly**: the aggregation, arrangement and bonding together of a set of components to form a tight junction, an occluding cell-cell junction that is composed of a branching network of sealing strands that completely encircles the apical end of each cell in an epithelial sheet.	PECAM1; ARL2; CLDN5; WNT11; CLDN3; APC; CLDN6; CLDN22; SLC39A9; OCLN; OCEL1; CLDN34; CLDN11; PAK2; CLDN23; CLDN16; CLDN14; ESAM; CLDN2; MARVELD3; CLDN1; CLDN9; CLDN7; RAB13; CLDN10; ECT2; DLG1; CLDN4; CDH5; CLDN25; CLDN19; MARVELD2; AFDN; SRF; CLDN24; STRN; MPP7; RAMP2; MICALL2; GRHL2; TBCD; POF1B; CLDN20; CLDN18; CLDN17; CLDN8; CLDN15; FRMPD2; PARD3, PDCD6IP
**Apical junction assembly:** the formation of an apical junction, a functional unit located near the cell apex at the points of contact between epithelial cells composed of tight junction, zonula adherens junction and desmosomes, by the aggregation, arrangement and bonding together of its constituents.	FBF1; RHOA; CTNNA1; WDR1; PKN2; RHOC; VCL
**Cell-cell junction assembly:** the aggregation, arrangement and bonding together of a set of components to form a junction between cells.	CLDN5; TRPV4; CDH24; CDH18; GJA4; CDH9; FSCN1; FLCN; TLN1; CDH19; CDH22; CDH10; TLN2; CDH17; DSG1; CDH7; CDH5; LIM2; CDH3; CDH2; TJP1; CDH4; NR1H4; CDH26; CDH20; GJC1; PKP4; HDAC7; CDH1; CDH15; CDH13; CDH12; CDH11; CDH8; CDH6; PARD6B
**Tight junction assembly:** a cellular process that results in the aggregation, arrangement and bonding together of a set of components to form a tight junction. A tight junction seals cells together in an epithelium in a way that prevents even small molecules from leaking from one side of the sheet to the other.	CLDN5; CLDN11; PATJ; MPDZ; EPHB2; CLDN12 CDHR3; ACTG1
**Adherens junction assembly:** the aggregation, arrangement and bonding together of a set of components to form an adherens junction. An adherens junction is a cell-cell junction composed	FER; HIPK1; PIP5K1C; CTNNB1; SMAD7; ZNF703, PAK2; ACTB; RAMP2; TBCD; VCL; JAM3

of the epithelial cadherin-catenin complex at which the cytoplasmic face of the plasma membrane is attached to actin filaments.	
**Tricellular tight junction assembly:** the aggregation, arrangement and bonding together of a set of components to form a tricellular tight junction	LSR; ILDR1
**Desmosome assembly**: a cellular process that results in the aggregation, arrangement and bonding together of a set of components to form a desmosome. A desmosome is a patch-like intercellular junction found in vertebrate tissues, consisting of parallel zones of 2 cell membranes, separated by a space of 25-35 nm, and having dense fibrillar plaques in subjacent cytoplasm. Desmosomes link two cells together; hemidesmosomes attach one cell to the extracellular matrix.	PKP2; PKP3; PRKCA; JUP
**Gap junction assembly**: assembly of gap junctions which serve as direct connections between the cytoplasms of adjacent cells. They provide open channels through the plasma membrane, allowing ions and small molecules (less than approximately a thousand daltons) to diffuse freely between neighboring cells, but preventing the passage of proteins and nucleic acids.	CTNNA1; GJB6; GJA1; GJD3; GJB1; GJB2; GJC1; GJA5
**Filtration diaphragm assembly:** the aggregation, arrangement and bonding together of a set of components to form a filtration diaphragm, a specialized cell-cell junction found between the cells of the excretory system, which provides a barrier for filtration of blood or hemolymph.	FKRP
**Slit diaphragm assembly**: The aggregation, arrangement and bonding together of a set of components to form a slit diaphragm, specialized cell-cell junction found between interdigitating foot processes of glomerular epithelium (the podocytes) in the vertebrate kidney, which is adapted for facilitating glomerular filtration.	PTPRO; NPHS1
**Paranodal junction assembly:** Formation of the junction between an axon and the glial cell that forms the myelin sheath. Paranodal junctions form at each paranode, i.e. at the ends of the unmyelinated nodes of Ranvier.	EPB41L3; CNTNAP1; GNPAT; UGT8; CD9; ANK2
**Zonula adherens assembly:** Assembly of the zonula adherens, a cell-cell adherens junction which forms a continuous belt near the apex of epithelial cells.	DLG5

*Adapted from https://amigo.geneontology.org/amigo/term/GO:0007043; **The same gene can be present in multiple classes

Supplementary Table 2. Genes excluded from analysis due to being overall expressed in less than 70% of foreskins from participant individuals.

Gene	n/N (%) expressing gene overall N=139	n/N (%) expressing gene, South Africa N=68	n/N (%) expressing gene, Uganda N=71	p-value[1]
CLDN18	1 (0.7%)	1 (1.5%)	0 (0%)	0.49
LIM2	1 (0.7%)	1 (1.5%)	0 (0%)	0.49
CLDN24	4 (2.9%)	1 (1.5%)	3 (4.2%)	0.62
CLDN2	5 (3.6%)	3 (4.4%)	2 (2.8%)	0.68
CLDN20	6 (4.3%)	6 (8.8%)	0 (0%)	0.01
CLDN14	15 (10.8%)	10 (14.7%)	5 (7.0%)	0.18
FRMPD2	17 (12.2%)	8 (11.8%)	9 (12.7%)	1.00
IGSF5	18 (12.9%)	12 (17.6%)	6 (8.5%)	0.13
C1QTNF5	19 (13.7%)	11 (16.2%)	8 (11.3%)	0.46
CLDN22	20 (14.4%)	10 (14.7%)	10 (14.1%)	1.00
CLDN25	35 (25.2%)	16 (23.5%)	19 (26.8%)	0.70
CLDN6	61 (43.9%)	29 (42.6%)	32 (45.1%)	0.89
CLDN9	63 (45.3%)	37 (54.4%)	26 (36.6%)	0.04
ILDR2	93 (66.9%)	48 (70.6%)	45 (63.3%)	0.38

[1]P-value for comparison of proportions expressing gene between South Africa and Uganda participants, generated using Fisher's exact test.

Supplementary Table 3. Effect of oral PrEP on tight junction gene expression, all participants.

Gene	PrEP = None	PrEP = FTC-TDF	PrEP = FTC-TAF
TRAF4	3108 (2777, 3486)	3014 (2423, 3504)	2864 (2269, 3442)
RAP2B	3211 (917, 3396)	2929 (1312, 3325)	2916 (650, 3411)
CCND1	2960 (2502, 3516)	2699 (1797, 3108)	3094 (1863, 3401)
NPHP4	3131 (967, 3362)	2734 (485, 3321)	2945 (1557, 3606)
TJAP1	3287 (3110, 3506)	2857 (2138, 3422)	2680 (926, 3287)
ACTB	2824 (2636, 3284)	2817 (2182, 3436)	2950 (2560, 3435)
CRB3	2876 (2658, 3186)	2872 (2236, 3443)	2839 (2555, 3601)
PRKCZ	3281 (354, 3523)	2699 (330, 3189)	2997 (393, 3376)
CDH5	3073 (2660, 3461)	2632 (2081, 3205)	2970 (2444, 3480)
ATP7B	2834 (2491, 3235)	2775 (2125, 3611)	2786 (2188, 3355)
NPHP1	2992 (2555, 3155)	2786 (2205, 3447)	2765 (2182, 3505)
RAPGEF2	2576 (2357, 3040)	2840 (2222, 3502)	2885 (2408, 3397)
CGNL1	2941 (1720, 3225)	2748 (2107, 3210)	2766 (2108, 3213)
ARHGEF2	3059 (2826, 3174)	2680 (2159, 3210)	2687 (2163, 3303)
RPGRIP1L	2691 (2212, 3153)	2790 (2342, 3312)	2687 (569, 3128)
MICALL2	2886 (593, 3206)	2716 (1588, 3265)	2651 (1737, 3322)
CLDN4	2519 (2184, 2909)	2699 (2228, 3133)	2731 (2192, 3384)
FZD5	2851 (2320, 3460)	2724 (2051, 3179)	2611 (1908, 3309)
TJP2	2976 (2185, 3276)	2790 (1449, 3393)	2488 (1686, 3186)
CLDN5	2879 (2116, 3406)	2592 (1899, 3085)	2627 (1505, 3037)
CLDN1	2524 (1908, 2940)	2653 (2236, 3273)	2674 (2122, 3289)
ILDR1	2766 (2312, 3071)	2633 (1990, 3172)	2534 (2009, 3344)
APC	2635 (2080, 2932)	2535 (2044, 3326)	2694 (2106, 3222)
POF1B	2320 (1222, 3123)	2753 (1982, 3350)	2531 (2141, 3432)
PRKCI	2385 (808, 2949)	2489 (1971, 3380)	2884 (2239, 3379)
ESAM	2669 (2275, 2877)	2462 (2000, 3287)	2560 (2095, 3270)
EPB41L4B	2483 (2120, 2929)	2511 (1996, 3027)	2629 (2048, 3286)
SHROOM2	2590 (2307, 3152)	2389 (1919, 3176)	2800 (2084, 3372)
JAM3	2523 (2160, 2916)	2364 (1886, 3300)	2581 (1955, 3164)
TJP3	2445 (2003, 2873)	2480 (2103, 2973)	2454 (2036, 2860)
CXADR	2721 (669, 3138)	2388 (546, 2999)	2291 (402, 2960)
CTNNB1	2281 (2050, 2562)	2458 (1953, 3026)	2476 (2021, 3007)
RAP2C	2346 (1918, 3159)	2414 (1801, 3172)	2409 (1897, 3034)
PARD6A	2448 (2136, 2759)	2190 (1845, 3011)	2288 (1784, 2853)
OCLN	2130 (1647, 2710)	2323 (1696, 2739)	2380 (1906, 2775)
ADCYAP1R1	2758 (2125, 3303)	2206 (1129, 2959)	2292 (1342, 2891)
JAM2	2415 (1839, 2575)	2057 (1704, 2806)	2270 (1830, 2691)
USP53	2208 (1692, 3245)	2161 (1726, 3320)	2345 (1904, 2943)
PLXDC1	2415 (1788, 3065)	2210 (1648, 2720)	2320 (1740, 2886)
PDCD6IP	2136 (1720, 2344)	2169 (1640, 2789)	2227 (1767, 2768)
CCDC85C	2119 (1989, 2342)	2213 (1723, 2726)	2273 (1773, 2632)
SAPCD2	2506 (2166, 2943)	2053 (1443, 2521)	2185 (1635, 2929)
LIN7C	2217 (1932, 2650)	2219 (1711, 2872)	2169 (1716, 2899)
BVES	2218 (1299, 2515)	2084 (1159, 2681)	2209 (1433, 2819)
PARD3	2186 (1749, 2521)	2169 (1714, 2447)	2080 (1761, 2608)
EPCAM	2342 (1495, 2762)	2341 (659, 3119)	1841 (618, 2791)
PARD3B	2037 (1456, 2310)	1971 (1455, 2792)	2119 (1730, 2638)
EPHA2	2249 (1131, 2906)	1882 (1354, 2547)	2140 (1339, 2709)
CLDN10	1161 (740, 2988)	2267 (1055, 3131)	1983 (690, 2880)
RIGI	1847 (1420, 2239)	1993 (1137, 2687)	1854 (1053, 2695)
CLDN17	2794 (1103, 3237)	1726 (607, 2514)	1838 (854, 2695)
MXRA8	2702 (674, 3483)	1555 (492, 2968)	1755 (472, 2940)

WNK4	1221 (338, 2548)	1931 (385, 2663)	2046 (869, 2752)
TGFBR1	2004 (1370, 2507)	1826 (1428, 2691)	1878 (1550, 2564)
F11R	1665 (1481, 2007)	1821 (1343, 2415)	1937 (1529, 2449)
VAPA	1827 (1294, 2148)	1632 (1221, 2411)	1867 (1417, 2328)
AOC1	1698 (1093, 2669)	1466 (716, 2851)	2058 (1094, 3014)
CLDN19	2588 (663, 3274)	2035 (676, 2934)	1145 (328, 2807)
RAB13	1836 (1651, 1947)	1776 (1290, 2426)	1754 (1337, 2253)
AMOT	1843 (1264, 2120)	1710 (1124, 2393)	1800 (1231, 2453)
PALS1	1748 (1307, 2346)	1687 (1169, 2559)	1801 (1352, 2352)
AMOTL1	1626 (1380, 2179)	1688 (1312, 2357)	1757 (1523, 2291)
CLDN12	1646 (743, 2490)	1700 (897, 2409)	1628 (1212, 2505)
MARVELD2	1637 (1323, 1927)	1553 (1143, 2207)	1725 (1300, 2174)
PARD6B	2246 (927, 3148)	1843 (518, 3123)	1436 (354, 2993)
CLDN16	1747 (846, 2713)	1644 (953, 2297)	1597 (842, 2315)
CLDN15	1663 (1494, 1975)	1655 (1128, 2511)	1568 (1027, 2309)
SYMPK	1674 (1410, 1837)	1568 (1245, 2264)	1587 (1263, 2113)
PARD6G	1527 (1206, 1774)	1612 (1134, 2077)	1590 (1263, 2236)
CLDN11	1445 (752, 2698)	1550 (758, 2839)	1630 (684, 2498)
LIN7B	1636 (1383, 2103)	1561 (1266, 2011)	1510 (1053, 2141)
CLDN8	1749 (521, 2963)	1540 (608, 2753)	1601 (473, 2808)
CLDN23	1491 (1204, 2567)	1328 (767, 1983)	1674 (765, 2369)
AFDN	1583 (911, 2140)	1528 (1035, 1992)	1546 (1111, 2055)
MAGI2	844 (287, 3204)	1039 (398, 2970)	2338 (422, 3233)
CLDN7	1394 (415, 1990)	1422 (641, 2854)	1480 (511, 2376)
LSR	1560 (1179, 1965)	1415 (1088, 2013)	1463 (1244, 2143)
CYTH2	1457 (1039, 1648)	1404 (973, 1850)	1348 (1061, 1994)
VASP	1306 (1049, 1704)	1310 (964, 1839)	1486 (1141, 1877)
PMP22	1106 (437, 1522)	1359 (785, 1766)	1414 (942, 1926)
LIN7A	1295 (173, 2314)	1681 (523, 2898)	1211 (413, 2686)
ECT2	1269 (393, 2127)	1409 (548, 2092)	1325 (790, 2010)
EPPK1	982 (717, 2151)	1571 (594, 2808)	1312 (569, 2138)
CNTNAP1	1295 (768, 1665)	1343 (800, 2046)	1255 (810, 2084)
CYTH1	1327 (1055, 1514)	1368 (919, 1714)	1264 (962, 1900)
WNK3	972 (413, 1889)	1644 (556, 2842)	1281 (420, 2641)
CLMP	1212 (765, 1759)	1286 (625, 2106)	1239 (620, 1847)
MTDH	1289 (721, 1545)	1200 (744, 1791)	1247 (850, 1740)
JAML	1201 (1088, 1673)	1334 (715, 1927)	1236 (653, 1999)
MAPK15	887 (387, 2858)	1188 (362, 2896)	1489 (532, 2770)
SH3BP1	455 (171, 3286)	1153 (256, 3157)	1951 (519, 3118)
MPDZ	1138 (571, 1429)	1299 (833, 1984)	1184 (879, 1698)
TBCD	1272 (906, 1478)	1202 (908, 1695)	1170 (932, 1642)
TJP1	1080 (680, 1861)	1141 (759, 1776)	1266 (854, 1756)
OCEL1	1175 (888, 1465)	1072 (794, 1714)	1198 (758, 2001)
CLDN3	467 (1, 2103)	916 (2, 2732)	1210 (151, 2407)
ASH1L	1966 (251, 3395)	1104 (295, 3091)	1048 (329, 3228)
MAGI3	1147 (677, 1560)	957 (548, 1725)	1181 (601, 1618)
CGN	941 (554, 1353)	1036 (668, 1543)	1078 (674, 1484)
GJA1	579 (188, 1671)	929 (497, 1560)	946 (503, 1475)
STRN	2132 (757, 3230)	1151 (472, 2894)	692 (325, 2633)
MPP7	1020 (471, 2114)	871 (463, 1601)	853 (418, 1362)
SYNPO	805 (463, 1219)	958 (575, 1729)	823 (539, 1516)
CDK4	863 (502, 1059)	863 (569, 1312)	941 (651, 1310)
DLG1	949 (363, 2152)	854 (408, 1581)	738 (421, 1351)
ANK3	547 (173, 1480)	806 (368, 2801)	962 (455, 3078)

SIPA1L3	700 (557, 883)	885 (506, 1369)	785 (398, 1526)
MARVELD3	722 (242, 996)	692 (314, 1890)	1018 (300, 2023)
FRMD4B	1244 (567, 3328)	738 (322, 1692)	728 (340, 1583)
MAGI1	706 (274, 3323)	515 (224, 2828)	909 (353, 3247)
PATJ	417 (205, 2613)	884 (431, 2922)	682 (226, 2865)
NFASC	923 (470, 3193)	637 (308, 2574)	734 (361, 3010)
FRMD4A	667 (335, 3474)	633 (197, 2612)	911 (326, 2956)
AMOTL2	392 (287, 792)	705 (353, 1426)	819 (439, 1569)
UBN1	501 (248, 3188)	491 (152, 2544)	719 (278, 2877)
CYTH3	522 (370, 736)	662 (387, 1125)	651 (490, 1060)
DLG3	469 (190, 901)	546 (296, 1787)	690 (298, 1082)
ARHGAP17	539 (362, 751)	588 (330, 1392)	582 (338, 1150)

Figures show median expression (with interquartile range in parentheses) by trial arm. Results are ordered by median expression level (high to low) among all participants. P-values (not shown) for differences between trial arms were calculated from Kruskal-Wallis test and adjusted for multiple testing using the false discovery rate (FDR), all FDR-adjusted p-values were >0.5.

Supplementary Table 4. Effect of oral PrEP on tight junction gene expression, South Africa participants.

Gene	PrEP = None	PrEP = FTC-TDF	PrEP = FTC-TAF
CRB3	2864 (2555, 3208)	2939 (1996, 3580)	3319 (2484, 3796)
ATP7B	2878 (2491, 3235)	3085 (2071, 3902)	2934 (2172, 3525)
JAM3	2442 (2160, 3034)	2701 (1780, 3509)	3030 (2156, 3405)
ACTB	3029 (2796, 3284)	2763 (2095, 3787)	3021 (2231, 3651)
ESAM	2666 (2363, 2980)	2912 (2047, 3758)	3136 (2167, 3393)
CCND1	3078 (2719, 3579)	2604 (580, 3005)	3175 (1949, 3459)
RAPGEF2	2416 (2272, 2607)	2840 (2105, 3616)	2993 (2364, 3584)
CGNL1	2451 (1534, 3424)	2748 (1713, 3171)	2986 (1929, 3328)
TRAF4	3011 (2777, 3153)	2782 (2288, 3841)	2792 (474, 3429)
SHROOM2	2729 (2299, 3152)	2445 (1977, 3552)	3132 (2341, 3943)
NPHP1	2985 (2459, 3071)	2711 (1711, 3676)	2902 (1984, 3636)
CDH5	2820 (2574, 3330)	2362 (549, 3359)	3371 (1765, 3555)
NPHP4	2747 (895, 3317)	2734 (707, 3392)	3240 (511, 3812)
PARD6A	2706 (2372, 2950)	2727 (2059, 3486)	2770 (2206, 3631)
ARHGEF2	3019 (2932, 3501)	2621 (1049, 3269)	2910 (547, 3901)
TJAP1	3313 (3208, 3533)	2524 (326, 3007)	2428 (254, 3455)
APC	2173 (2011, 2893)	2535 (1930, 3401)	2965 (2278, 3458)
ILDR1	2641 (2328, 2823)	2518 (1587, 3272)	2873 (2056, 3798)
RAP2B	3225 (3142, 3396)	2462 (673, 3055)	2013 (450, 3348)
RPGRIP1L	2950 (2351, 3153)	2766 (1840, 3799)	2377 (346, 3446)
LIN7C	2289 (1984, 2780)	2526 (1673, 2939)	2818 (1983, 3223)
TJP2	2976 (2530, 3060)	2593 (1488, 3602)	2653 (2020, 3197)
CLDN5	3021 (2186, 3406)	2415 (1577, 3287)	2748 (571, 3238)
CLDN1	2666 (2058, 3199)	2486 (2046, 3331)	2835 (2192, 3405)
CLDN4	2214 (1909, 2687)	2700 (1926, 3322)	2654 (1931, 3398)
EPB41L4B	2483 (1922, 2597)	2361 (1755, 3163)	2777 (2022, 3405)
RAP2C	2282 (1909, 3307)	2767 (1735, 3286)	2663 (1971, 3242)
POF1B	2237 (462, 2555)	2556 (1821, 3350)	2874 (2107, 3507)
CTNNB1	2206 (2006, 2368)	2148 (1839, 3332)	2854 (2118, 3141)
PLXDC1	2185 (1547, 3031)	2336 (1705, 2821)	2642 (1781, 3279)
USP53	1797 (1627, 3300)	2463 (1598, 3416)	2632 (2082, 3281)
FZD5	2536 (1312, 3180)	2343 (1634, 3298)	2678 (1466, 3454)
PRKCI	2370 (335, 2484)	2489 (2170, 3515)	2869 (2114, 3506)
TJP3	2435 (1692, 2842)	2367 (1716, 3070)	2709 (1952, 3210)
JAM2	2415 (1613, 2538)	2430 (1748, 3232)	2489 (1973, 2889)
ADCYAP1R1	2758 (2448, 3238)	2206 (955, 3098)	2135 (866, 3152)
CCDC85C	2153 (1974, 2719)	2265 (1450, 3021)	2522 (1833, 2760)
SAPCD2	2654 (2260, 3374)	2198 (1141, 2795)	2905 (2007, 3499)
CXADR	2567 (753, 2988)	2458 (1187, 3175)	1855 (328, 2942)
OCLN	1811 (1648, 2451)	2239 (1437, 2994)	2422 (1856, 3224)
PDCD6IP	1780 (1644, 2344)	2169 (1540, 3043)	2390 (1885, 2920)
BVES	2218 (1618, 2412)	2131 (1159, 3190)	2182 (1108, 2964)
PARD6B	2647 (1422, 3296)	2352 (563, 3338)	1635 (314, 2913)
CLDN10	2088 (921, 3314)	2352 (726, 3152)	1737 (595, 3127)
TGFBR1	2076 (1554, 2507)	2094 (1219, 2875)	2288 (1701, 2869)
PARD3B	2037 (1285, 2163)	1984 (1425, 2876)	2299 (1892, 2891)
PARD3	1855 (1248, 2360)	1969 (1563, 2448)	2083 (1777, 2595)
AOC1	1698 (1197, 2855)	1793 (1042, 3238)	2527 (1693, 3356)
F11R	1486 (1311, 1828)	2051 (1289, 2481)	2410 (1558, 2795)
VAPA	1563 (1174, 2088)	2107 (1015, 2473)	2023 (1472, 2404)

CLDN15	1933 (1554, 2218)	1737 (1263, 2638)	2162 (1547, 2365)
MICALL2	599 (257, 2480)	2165 (434, 3002)	2170 (455, 3389)
LSR	1606 (1247, 2024)	1958 (865, 2331)	1987 (1555, 2452)
RIGI	1633 (1420, 2446)	1602 (1034, 2687)	2182 (1090, 2786)
RAB13	1694 (1535, 1872)	2348 (1147, 2724)	1955 (1663, 2941)
LIN7B	1954 (1609, 2192)	1765 (1266, 2269)	1934 (1350, 2240)
EPHA2	2154 (1279, 3030)	1822 (1195, 2923)	2127 (1455, 2929)
CNTNAP1	1423 (852, 1840)	1948 (1287, 2696)	1914 (1331, 2802)
SYMPK	1701 (1430, 1864)	1661 (1228, 2376)	1993 (1521, 2344)
CLDN16	2252 (1648, 2913)	1644 (934, 2159)	1974 (907, 2713)
PALS1	1467 (1307, 2468)	1719 (1134, 2536)	1833 (1341, 2433)
WNK4	1221 (338, 2856)	1998 (474, 2517)	2018 (793, 2813)
AMOTL1	1406 (1175, 1760)	1608 (1061, 2483)	2139 (1514, 2426)
CLDN17	1981 (375, 3151)	1699 (465, 2398)	2165 (1273, 2846)
CLDN12	1259 (547, 2598)	1719 (838, 2567)	1764 (1354, 2640)
CLDN11	1816 (802, 3153)	1480 (805, 2531)	1834 (740, 2501)
AMOT	1606 (1159, 2001)	1744 (1124, 2220)	1743 (1116, 2422)
AFDN	1530 (986, 2181)	1613 (840, 2350)	1900 (1089, 2228)
CLDN19	2519 (72, 3274)	1856 (622, 2936)	1262 (366, 2762)
VASP	1533 (1047, 1932)	1665 (929, 2136)	1763 (1461, 2269)
EPCAM	2342 (887, 3004)	1853 (439, 2914)	1560 (670, 2511)
CLDN8	2869 (662, 3245)	1540 (548, 2544)	2032 (612, 3055)
CYTH2	1325 (1039, 1697)	1528 (957, 2045)	1796 (1212, 2265)
MARVELD2	1511 (1144, 1807)	1442 (1049, 2207)	1900 (1346, 2190)
OCEL1	1436 (706, 1777)	1341 (891, 2007)	1922 (1221, 2247)
PMP22	881 (437, 1568)	1682 (947, 2181)	1727 (1195, 2153)
JAML	1660 (1302, 2354)	1344 (837, 2367)	1796 (1135, 2537)
PARD6G	1496 (1259, 1717)	1552 (932, 2198)	1783 (1372, 2346)
CLDN23	1209 (574, 2078)	1328 (910, 1983)	1702 (769, 2439)
CLMP	1192 (538, 1753)	1536 (831, 2607)	1595 (1049, 2039)
CYTH1	1228 (949, 1512)	1382 (868, 1950)	1662 (994, 2026)
PRKCZ	3171 (354, 3399)	804 (314, 2839)	1161 (323, 3398)
ECT2	692 (150, 2043)	1563 (911, 2271)	1314 (767, 2050)
EPPK1	1353 (736, 3052)	1417 (412, 2732)	1518 (584, 2119)
MPDZ	1175 (654, 1429)	1590 (731, 2080)	1455 (1018, 2003)
TBCD	1350 (750, 1478)	1445 (824, 1884)	1528 (1142, 1944)
CLDN7	1043 (415, 1990)	1722 (713, 2723)	1121 (440, 2185)
LIN7A	738 (37, 1525)	1681 (996, 2648)	1334 (553, 2544)
TJP1	877 (680, 1857)	1265 (563, 1782)	1350 (835, 1920)
MAGI2	728 (202, 2988)	1032 (398, 2665)	2371 (623, 3364)
MTDH	784 (721, 1545)	1569 (668, 1791)	1285 (856, 1812)
MAGI3	1036 (204, 1540)	1145 (548, 1929)	1336 (774, 1755)
SYNPO	721 (435, 1192)	1370 (744, 2180)	1472 (749, 1773)
CLDN3	1453 (1, 3491)	1209 (2, 3050)	1224 (221, 2359)
MAGI1	2800 (460, 3485)	1202 (273, 2934)	1161 (381, 3360)
STRN	2982 (1044, 3291)	883 (448, 2750)	1005 (373, 2892)
WNK3	861 (70, 1657)	1532 (556, 2603)	1145 (398, 2501)
MXRA8	2992 (891, 3441)	1039 (418, 2210)	1145 (435, 2499)
ASH1L	3214 (260, 3479)	1297 (501, 2942)	692 (273, 3242)
CDK4	1015 (442, 1171)	863 (475, 1600)	1255 (753, 1582)
CGN	836 (400, 1448)	1059 (327, 1760)	1131 (634, 1555)
GJA1	343 (127, 1564)	1071 (497, 2388)	1195 (580, 1718)
MPP7	775 (337, 1511)	1051 (668, 2429)	985 (584, 1427)
MAPK15	887 (350, 2544)	715 (362, 2302)	1243 (548, 2957)

AMOTL2	334 (193, 519)	1070 (596, 1579)	1138 (623, 1670)
ARHGAP17	516 (362, 702)	972 (331, 1509)	950 (552, 1308)
FRMD4A	903 (376, 3474)	700 (197, 1862)	985 (265, 1675)
SIPA1L3	808 (499, 1085)	1041 (506, 1640)	1075 (336, 1739)
DLG1	925 (238, 2856)	1059 (465, 1794)	752 (446, 1684)
NFASC	803 (393, 3319)	952 (360, 2610)	801 (483, 3020)
MARVELD3	823 (555, 1005)	548 (308, 1688)	1139 (750, 1754)
ANK3	573 (185, 3129)	945 (382, 2872)	841 (399, 3029)
PATJ	1940 (339, 3396)	1053 (441, 2850)	726 (423, 2972)
FRMD4B	2050 (567, 3385)	855 (388, 2818)	732 (292, 2833)
DLG3	514 (159, 2710)	957 (326, 2768)	776 (435, 1411)
CYTH3	517 (148, 878)	907 (260, 1571)	863 (582, 1116)
UBN1	1991 (330, 3336)	655 (245, 2544)	786 (616, 2069)
SH3BP1	455 (190, 2786)	765 (446, 2604)	783 (398, 1244)

Figures show median expression (with interquartile range in parentheses) by trial arm. Results are ordered by median expression level (high to low) among all South Africa participants. P-values (not shown) for differences between trial arms were calculated from Kruskal-Wallis test and adjusted for multiple testing using the false discovery rate (FDR), all FDR-adjusted p-values were >0.5.

Supplementary Table 5. Effect of oral PrEP on tight junction gene expression, Uganda participants.

Gene	PrEP = None	PrEP = FTC-TDF	PrEP = FTC-TAF
RAP2B	2939 (178, 3565)	3186 (2683, 3396)	3162 (2573, 3420)
PRKCZ	3464 (824, 3613)	3086 (377, 3445)	3135 (750, 3383)
TRAF4	3297 (2773, 3546)	3053 (2850, 3277)	2877 (2483, 3536)
TJAP1	3268 (2952, 3495)	3081 (2592, 3557)	2785 (2497, 3240)
SH3BP1	1699 (136, 3286)	3057 (237, 3439)	2991 (2007, 3370)
NPHP4	3183 (1127, 3582)	2825 (328, 3308)	2931 (2283, 3362)
CCND1	2820 (1418, 3488)	2878 (2240, 3420)	3047 (1862, 3360)
MICALL2	3126 (2875, 3332)	2984 (2471, 3356)	2701 (2275, 2981)
CDH5	3296 (2712, 3581)	2784 (2437, 3126)	2854 (2500, 3183)
ACTB	2664 (2561, 3175)	2852 (2410, 3244)	2887 (2634, 3223)
NPHP1	3026 (2627, 3382)	2828 (2257, 3440)	2730 (2320, 3219)
CRB3	2887 (2662, 3186)	2864 (2458, 3196)	2735 (2597, 3097)
FZD5	3243 (2657, 3660)	2817 (2474, 3171)	2567 (1982, 3237)
RPGRIP1L	2621 (940, 3151)	2861 (2436, 3253)	2731 (1962, 3072)
CGNL1	2941 (2324, 3171)	2756 (2213, 3239)	2744 (2173, 3107)
RAPGEF2	2961 (2482, 3327)	2825 (2414, 3154)	2755 (2412, 3343)
CLDN4	2833 (2457, 2935)	2696 (2414, 3058)	2771 (2498, 3255)
ARHGEF2	3062 (2633, 3132)	2903 (2526, 3139)	2665 (2475, 2944)
ATP7B	2821 (2397, 3233)	2716 (2187, 3217)	2685 (2226, 3010)
MXRA8	2642 (504, 3492)	2831 (541, 3363)	2554 (540, 3300)
CLDN1	2462 (808, 2774)	2952 (2390, 3252)	2591 (2047, 3230)
PRKCI	2850 (2145, 3197)	2484 (1835, 2947)	2910 (2247, 3336)
POF1B	2620 (1930, 3316)	2766 (2389, 3366)	2383 (2139, 3233)
TJP2	2855 (1134, 3445)	2854 (1097, 3294)	2433 (518, 3166)
CLDN5	2720 (1971, 3477)	2596 (2110, 3041)	2594 (2097, 2899)
ILDR1	2839 (2295, 3397)	2637 (2057, 3105)	2373 (1984, 2915)
APC	2724 (2224, 2953)	2528 (2177, 3041)	2529 (2061, 2958)
CXADR	2828 (618, 3278)	2267 (379, 2955)	2508 (1018, 2974)
EPB41L4B	2567 (2123, 3123)	2535 (2223, 2912)	2357 (2052, 3045)
TJP3	2480 (2116, 2873)	2505 (2246, 2950)	2438 (2220, 2778)
ESAM	2710 (2267, 2877)	2417 (1967, 2761)	2430 (2041, 2610)
EPCAM	2289 (1495, 2636)	2622 (1082, 3222)	2185 (503, 2935)
SHROOM2	2462 (2307, 3221)	2339 (1897, 2873)	2255 (2011, 3059)
CTNNB1	2362 (2166, 2780)	2469 (1967, 2873)	2198 (1954, 2822)
OCLN	2443 (1419, 2879)	2328 (1960, 2715)	2314 (1957, 2667)
ADCYAP1R1	2661 (1550, 3303)	2206 (1454, 2943)	2330 (1556, 2781)
JAM3	2549 (2132, 2718)	2135 (1950, 2775)	2280 (1913, 2692)
RAP2C	2346 (1983, 2848)	2341 (1986, 3025)	2165 (1847, 2786)
PARD3	2413 (2152, 2730)	2251 (2051, 2437)	2037 (1750, 2668)
PLXDC1	2604 (1832, 3073)	2169 (1584, 2429)	2103 (1660, 2614)
PDCD6IP	2221 (1810, 2656)	2191 (1811, 2693)	2090 (1670, 2667)
USP53	2453 (1900, 3021)	1975 (1749, 3022)	2171 (1837, 2528)
LIN7C	2096 (1826, 2377)	2192 (1713, 2688)	2018 (1608, 2457)
MAGI2	1781 (477, 3243)	1693 (378, 3062)	2305 (287, 3249)
PARD6A	2413 (1933, 2671)	2100 (1699, 2587)	1839 (1559, 2361)
EPHA2	2249 (931, 2906)	2022 (1391, 2376)	2152 (1219, 2486)
CCDC85C	2095 (2004, 2303)	2200 (1865, 2384)	1965 (1728, 2440)
SAPCD2	2478 (1799, 2889)	2029 (1677, 2298)	1968 (1533, 2341)
JAM2	2360 (2068, 2784)	1953 (1652, 2744)	2032 (1743, 2443)
BVES	1971 (1118, 2623)	1975 (1136, 2408)	2236 (1552, 2738)
CLDN17	2829 (1519, 3532)	1978 (770, 2766)	1660 (695, 2693)
RIGI	2068 (1351, 2239)	2109 (1248, 2690)	1752 (1000, 2309)

CLDN10	1127 (178, 1982)	2102 (1209, 2796)	2164 (819, 2675)
PARD3B	2128 (1613, 2592)	1949 (1583, 2702)	1989 (1562, 2333)
WNK4	1699 (246, 2548)	1857 (66, 2856)	2093 (1041, 2757)
AMOT	1960 (1408, 2305)	1526 (1124, 2579)	1801 (1330, 2490)
TGFBR1	1937 (1147, 2569)	1764 (1447, 2648)	1801 (1017, 2321)
CLDN19	2588 (1226, 3317)	2334 (735, 2928)	1115 (305, 2878)
F11R	1813 (1583, 2139)	1808 (1628, 2275)	1698 (1515, 2129)
VAPA	1845 (1408, 2343)	1631 (1306, 2342)	1769 (1193, 2258)
PALS1	1814 (1349, 2341)	1658 (1235, 2619)	1686 (1381, 2242)
MARVELD2	1775 (1449, 1951)	1792 (1365, 2180)	1592 (1174, 1903)
AMOTL1	1828 (1581, 2348)	1748 (1361, 2194)	1652 (1550, 2046)
RAB13	1875 (1832, 2169)	1657 (1375, 2147)	1503 (1308, 1794)
MAPK15	949 (439, 2969)	1525 (332, 3076)	2334 (434, 2758)
PARD6G	1603 (1177, 1903)	1637 (1425, 1998)	1481 (1249, 2008)
CLDN23	2176 (1491, 2710)	1317 (766, 2058)	1576 (759, 2374)
WNK3	1063 (510, 2645)	1714 (559, 2875)	1551 (419, 2769)
CLDN16	1113 (272, 2248)	1648 (1079, 2455)	1417 (816, 2179)
CLDN12	1646 (984, 2322)	1435 (919, 2372)	1463 (1114, 2320)
CLDN7	1394 (287, 2453)	1209 (467, 2982)	1605 (696, 2679)
AOC1	1938 (929, 2669)	1308 (522, 2762)	1544 (402, 2657)
SYMPK	1515 (1410, 1766)	1567 (1255, 1771)	1347 (1094, 1652)
CLDN11	1172 (737, 2439)	1646 (670, 2924)	1232 (683, 2544)
AFDN	1605 (843, 2140)	1490 (1159, 1861)	1382 (1121, 1887)
LIN7B	1458 (1162, 1776)	1479 (1157, 1721)	1260 (731, 1815)
CLDN15	1520 (1186, 1787)	1385 (921, 2024)	1243 (846, 1767)
LSR	1407 (1179, 1854)	1359 (1147, 1680)	1321 (1086, 1475)
EPPK1	932 (502, 1685)	1590 (676, 2946)	1220 (497, 2441)
CYTH2	1517 (1105, 1648)	1381 (1071, 1673)	1226 (1009, 1520)
LIN7A	1843 (567, 2621)	1566 (308, 3050)	819 (384, 2901)
VASP	1191 (1049, 1544)	1304 (1034, 1503)	1183 (1050, 1513)
CLDN8	1074 (521, 2143)	1642 (627, 2827)	824 (419, 2197)
MTDH	1353 (764, 1634)	1185 (839, 1838)	1195 (850, 1463)
CYTH1	1364 (1188, 1609)	1326 (985, 1586)	1120 (953, 1683)
ECT2	1438 (1213, 2877)	1037 (431, 2058)	1340 (784, 1808)
PARD6B	1067 (456, 2826)	1220 (447, 3086)	1223 (431, 3090)
PMP22	1106 (465, 1490)	1157 (764, 1499)	1169 (868, 1533)
TJP1	1256 (620, 1863)	1104 (776, 1576)	1142 (921, 1533)
CNTNAP1	1101 (768, 1376)	1247 (675, 1408)	873 (695, 1242)
TBCD	1187 (1059, 1497)	1148 (982, 1330)	1026 (924, 1171)
ASH1L	626 (202, 3351)	939 (199, 3275)	2802 (332, 3225)
CLMP	1212 (858, 1781)	1022 (586, 1663)	957 (487, 1491)
JAML	1126 (945, 1169)	971 (558, 1820)	938 (616, 1554)
CGN	942 (713, 1266)	1006 (811, 1406)	1020 (690, 1418)
MPDZ	1138 (542, 1573)	1012 (850, 1740)	982 (845, 1568)
CLDN3	157 (34, 1078)	197 (1, 2283)	1195 (150, 2683)
OCEL1	1110 (970, 1251)	908 (738, 1388)	1004 (673, 1202)
MAGI3	1210 (926, 1885)	834 (524, 1655)	828 (588, 1478)
STRN	1069 (246, 3020)	1157 (669, 3137)	590 (208, 2493)
GJA1	707 (543, 1857)	825 (431, 1406)	866 (303, 1458)
ANK3	480 (142, 1089)	678 (366, 1690)	975 (598, 3198)
MPP7	1084 (631, 2914)	778 (313, 1402)	778 (401, 1363)
CDK4	814 (521, 1010)	864 (591, 1108)	733 (620, 999)
SIPA1L3	679 (632, 728)	839 (509, 1054)	763 (473, 1091)
SYNPO	932 (463, 1423)	775 (512, 1225)	680 (400, 881)

DLG1	949 (443, 2004)	571 (233, 1505)	723 (410, 1329)
FRMD4B	1183 (498, 2843)	536 (310, 928)	723 (388, 1251)
MARVELD3	439 (161, 985)	693 (345, 2994)	545 (139, 2327)
FRMD4A	563 (228, 3463)	525 (192, 3132)	652 (349, 3198)
CYTH3	587 (419, 736)	634 (438, 895)	597 (356, 896)
MAGI1	522 (130, 973)	413 (200, 1010)	840 (305, 3004)
NFASC	1011 (495, 3153)	560 (250, 2379)	637 (268, 3017)
PATJ	223 (124, 437)	848 (367, 2948)	678 (165, 2795)
ARHGAP17	636 (334, 804)	572 (321, 675)	454 (321, 758)
AMOTL2	551 (330, 920)	480 (264, 910)	657 (404, 1185)
DLG3	469 (210, 890)	401 (265, 1015)	432 (200, 851)
UBN1	393 (208, 771)	392 (127, 2529)	453 (177, 3180)

Figures show median expression (with interquartile range in parentheses) by trial arm. Results are ordered by median expression level (high to low) among all Uganda participants. P-values (not shown) for differences between trial arms were calculated from Kruskal-Wallis test and adjusted for multiple testing using the false discovery rate (FDR), all FDR-adjusted p-values were >0.5.

Supplementary Table 6. Foreskin cytokine genes excluded from analysis due to being detected in less than 70% of participant samples.

Gene	n/N (%) expressing gene overall, N=139	n/N (%) expressing gene, South Africa, N=68	n/N (%) expressing gene, Uganda, N=71	p-value[1]
IFNa	0 (0%)	0 (0%)	0 (0%)	--
IL2	5 (3.6%)	1 (1.5%)	4 (5.6%)	0.37
IL4	5 (3.6%)	4 (5.9%)	1 (1.4%)	0.20
IL31	28 (20.1%)	16 (23.5%)	12 (16.9%)	0.40
IL17A	54 (38.8%)	35 (51.5%)	19 (26.8%)	0.003
IL17F	60 (43.2%)	41 (60.3%)	19 (26.8%)	0.001
IFNγ	70 (50.4%)	31 (45.6%)	39 (54.9%)	0.31
IL13	78 (56.1%)	36 (52.9%)	42 (59.2%)	0.50

[1]P-value for comparison of proportions expressing gene between South Africa and Uganda participants, generated using Fisher's exact test.

Supplementary Table 7. Effect of oral PrEP on cytokine gene expression levels in tissue, overall and by country.

Gene	PrEP = FTC-TDF	PrEP = FTC-TAF	PrEP = None	p-value
**All participants**				
SMAD4	3114 (1069, 3298)	2765 (747, 3138)	2727 (583, 3255)	0.79
TNF	2317 (1945, 2723)	1848 (1215, 2524)	2455 (1572, 2880)	0.07
IL33	2281 (1612, 2662)	2076 (1424, 2880)	2202 (1605, 3044)	0.90
IL18	1874 (1695, 2664)	2011 (1603, 2730)	2282 (1597, 2718)	0.96
TGFβ1	2740 (547, 3259)	2570 (499, 3187)	1501 (532, 3090)	0.77
EGF	1545 (663, 2316)	2194 (1466, 2773)	1847.5 (851, 2834)	0.37
IL1β	1861 (293, 2762)	1802 (692, 2367)	2024 (811, 2802)	0.49
MMP9	1788 (619, 2746)	1653 (1204, 2811)	2081 (1229, 2884)	0.74
IL1α	1849 (589, 2996)	2222 (309, 2675)	1466 (498, 2637)	0.85
IL8	1014 (170, 2076)	1721 (2, 2788)	1692 (660, 2696)	0.64
VEGF	1758 (1239, 2425)	1649 (969, 2214)	1287 (753, 2265)	0.52
IL6	1206 (265, 2317)	1279 (156, 2522)	1588 (447, 2649)	0.85
IL10	859 (2, 2930)	1388 (3, 2318)	1158.5 (36, 2361)	0.89
**South African participants only**				
SMAD4	2998 (934, 3298)	2733 (814, 3138)	2372 (160, 3457)	0.72
IL33	2380 (1539, 2674)	2331 (1820, 3187)	2507 (1531, 3202)	0.92
MMP9	2447 (1844, 3251)	2262 (1421, 2904)	2245 (1353, 3077)	0.97
TNF	2202 (1187, 2413)	1781 (794, 2388)	2481 (1718, 2942)	0.10
IL18	1755 (1103, 2488)	1925 (1497, 2713)	2266 (1521.5, 2716)	0.71
EGF	1132 (430, 2197)	2226 (1205, 2773)	2094 (658, 3164)	0.41
VEGF	2396 (1743, 2705)	1980 (1268, 2465)	1472 (1036, 2574)	0.56
IL1α	1836 (601, 2750)	2222 (309, 2876)	1069 (350, 2390)	0.49
IL1β	1861 (831, 2468)	1135 (524, 2522)	1929 (677, 2796)	0.92
IL6	640.5 (2, 2062)	1113 (2, 2196)	2085 (54, 2886)	0.43
IL10	1609 (2, 3151)	1706 (138, 2453)	1217 (2, 2311)	0.86
IL8	1294 (227, 2658)	671 (2, 2253)	2065 (174, 2900)	0.38
TGFβ1	679 (278, 2553)	1273 (439, 2848)	736 (489, 1501)	0.69
**Ugandan participants only**				
TGFβ1	3120 (2659, 3650)	3113 (2362, 3333)	3005 (820, 3297)	0.65
SMAD4	3140 (1151, 3386)	2850 (302, 3161)	2853 (1087, 3181)	0.65
IL18	2215 (1820, 2688)	2225 (1749, 2803)	2295 (1633, 2720)	0.99
TNF	2420 (2134, 2880)	2047 (1305, 2604)	2414 (1556, 2884)	0.48
IL1α	2380 (283, 3409)	2069 (90, 2609)	2219 (523, 2878)	0.78
IL1β	1564 (39, 3138)	1941 (1020, 2339)	2120 (1368, 2895)	0.59
EGF	1833 (815, 3024)	2161 (1596, 2784)	1743 (885, 2827)	0.74
IL33	2019 (1612, 2614)	1863 (1302, 2456)	1952 (1599, 3025)	0.65
IL8	1014 (60, 182)	1995 (494, 3032)	1524 (841, 2642)	0.43
IL6	1383 (1160, 2532)	1801 (485, 2714)	1551 (486, 2380)	0.95
MMP9	973 (359, 1657)	1539 (805, 2246)	1776 (1031, 2761)	0.26
VEGF	1242 (998, 1676)	1255 (793, 1914)	1184 (470, 1815)	0.74
IL10	593 (2, 2203)	1183 (2, 2197)	1069 (133, 2452)	0.92

Median expression (with interquartile range in parentheses) by trial arm is shown. Results are ordered by median expression level (high to low) among all participants. P-values for differences between trial arms were calculated from Kruskal-Wallis test and adjusted for multiple testing using the false discovery rate (FDR), all FDR-adjusted p-values were >0.5.

Supplementary Table 8. Correlations of tight junctions and cytokines gene expression with drug concentrations in foreskin tissue.

TJ Genes	Correlation[1] with TDF-DP (p-value)	TJ Gene	Correlation[1] with FTC-TP (p-value)
BVES	0.23 (0.01)	*AOC1*	-0.22 (0.01)
MXRA8	0.2 (0.03)	*LIN7A*	0.21 (0.02)
LIN7B	-0.19 (0.03)	*CLDN7*	0.2 (0.02)
CXADR	-0.19 (0.04)	*MXRA8*	0.19 (0.03)
ESAM	-0.18 (0.05)	*WNK3*	0.18 (0.04)
AMOT	0.17 (0.07)	*FRMD4A*	-0.18 (0.05)
ARHGAP17	-0.16 (0.07)	*JAM3*	-0.16 (0.07)
SH3BP1	0.16 (0.08)	*ADCYAP1R1*	-0.16 (0.07)
MAPK15	0.15 (0.09)	*NFASC*	-0.16 (0.07)
CYTH3	-0.15 (0.09)	*LIN7B*	-0.15 (0.09)
CLMP	-0.15 (0.09)	*OCEL1*	-0.15 (0.09)
PARD6A	-0.14 (0.12)	*SH3BP1*	0.15 (0.11)
PARD6B	-0.14 (0.13)	*MAPK15*	0.14 (0.12)
CLDN23	0.13 (0.15)	*CLDN11*	0.14 (0.12)
RAB13	-0.13 (0.17)	*CLDN16*	0.14 (0.13)
OCEL1	-0.12 (0.18)	*CLMP*	-0.14 (0.14)
STRN	-0.12 (0.19)	*ESAM*	-0.13 (0.14)
TBCD	-0.12 (0.20)	*CCND1*	-0.13 (0.14)
MPP7	-0.11 (0.23)	*ECT2*	0.13 (0.15)
CDH5	0.11 (0.24)	*CLDN10*	-0.13 (0.16)
CLDN5	-0.11 (0.24)	*PRKCI*	-0.13 (0.17)
PARD3B	0.11 (0.24)	*JAM2*	-0.13 (0.17)
FRMD4B	-0.10 (0.25)	*EPCAM*	-0.12 (0.17)
LSR	-0.10 (0.26)	*ARHGAP17*	-0.12 (0.17)
NPHP4	0.10 (0.27)	*CYTH3*	-0.12 (0.19)
SYMPK	-0.10 (0.29)	*CNTNAP1*	-0.11 (0.21)
SYNPO	-0.09 (0.32)	*DLG3*	-0.11 (0.21)
JAM3	-0.09 (0.33)	*BVES*	0.11 (0.23)
CLDN10	-0.09 (0.33)	*PARD3*	0.11 (0.25)
PALS1	0.08 (0.35)	*CLDN5*	-0.1 (0.25)
MARVELD3	0.08 (0.36)	*SYNPO*	-0.1 (0.26)
PARD3	0.08 (0.36)	*RIGI*	0.09 (0.30)
ANK3	-0.08 (0.37)	*AMOT*	0.09 (0.31)
FZD5	-0.08 (0.38)	*SIPA1L3*	0.09 (0.31)
OCLN	0.08 (0.39)	*EPB41L4B*	0.09 (0.33)
EPB41L4B	0.08 (0.41)	*CXADR*	-0.09 (0.34)
CCDC85C	-0.07 (0.41)	*CLDN4*	0.08 (0.37)
CYTH1	-0.07 (0.42)	*LSR*	-0.08 (0.40)
TRAF4	-0.07 (0.42)	*CLDN1*	-0.08 (0.40)
TJAP1	0.07 (0.42)	*TBCD*	-0.08 (0.41)
WNK4	-0.07 (0.43)	*JAML*	0.07 (0.43)
CLDN19	-0.07 (0.43)	*WNK4*	-0.07 (0.45)
ILDR1	-0.07 (0.44)	*PARD6G*	0.07 (0.47)
EPHA2	-0.07 (0.44)	*ACTB*	-0.07 (0.47)
RAPGEF2	0.07 (0.44)	*PATJ*	0.07 (0.47)

F11R	0.07 (0.45)	*ILDR1*	-0.06 (0.48)
ACTB	-0.07 (0.45)	*NPHP1*	-0.06 (0.49)
CLDN15	-0.07 (0.46)	*RAPGEF2*	0.06 (0.50)
JAM2	-0.07 (0.46)	*USP53*	-0.06 (0.51)
TGFBR1	0.07 (0.46)	*MAGI3*	-0.06 (0.52)
GJA1	-0.07 (0.47)	*MAGI2*	0.06 (0.53)
EPCAM	-0.06 (0.49)	*PARD6A*	-0.06 (0.54)
ATP7B	0.06 (0.50)	*PMP22*	-0.06 (0.54)
CGN	-0.06 (0.50)	*RAB13*	-0.05 (0.56)
CLDN4	0.06 (0.51)	*CLDN23*	0.05 (0.58)
RIGI	0.06 (0.51)	*MPP7*	-0.05 (0.58)
MAGI1	0.06 (0.51)	*CLDN15*	0.05 (0.59)
DLG3	-0.06 (0.51)	*SYMPK*	-0.05 (0.60)
AOC1	-0.06 (0.51)	*UBN1*	-0.05 (0.61)
VASP	-0.06 (0.51)	*RPGRIP1L*	0.05 (0.62)
VAPA	0.06 (0.53)	*TGFBR1*	-0.04 (0.63)
ECT2	0.06 (0.53)	*ARHGEF2*	-0.04 (0.63)
CNTNAP1	-0.06 (0.54)	*MICALL2*	0.04 (0.64)
CYTH2	-0.05 (0.55)	*SAPCD2*	-0.04 (0.65)
MICALL2	0.05 (0.56)	*CYTH2*	-0.04 (0.67)
CLDN11	-0.05 (0.56)	*CCDC85C*	-0.04 (0.67)
CLDN17	0.05 (0.59)	*STRN*	0.04 (0.67)
EPPK1	-0.05 (0.59)	*PARD6B*	-0.04 (0.68)
MAGI2	0.05 (0.59)	*CLDN3*	0.04 (0.69)
MAGI3	-0.05 (0.6)	*ASH1L*	0.04 (0.69)
ASH1L	-0.05 (0.61)	*MARVELD2*	0.03 (0.72)
JAML	-0.05 (0.62)	*AMOTL1*	0.03 (0.72)
DLG1	-0.05 (0.62)	*F11R*	0.03 (0.73)
MARVELD2	0.04 (0.63)	*TJP3*	0.03 (0.73)
AMOTL1	0.04 (0.63)	*NPHP4*	0.03 (0.74)
AFDN	-0.04 (0.64)	*AFDN*	-0.03 (0.75)
CLDN12	0.04 (0.65)	*CDK4*	0.03 (0.75)
APC	0.04 (0.66)	*EPPK1*	0.03 (0.76)
PATJ	-0.04 (0.67)	*TJAP1*	-0.03 (0.76)
TJP3	-0.04 (0.67)	*PDCD6IP*	0.03 (0.77)
CLDN3	-0.04 (0.68)	*TJP2*	0.03 (0.78)
TJP2	-0.04 (0.69)	*FRMD4B*	-0.03 (0.78)
SAPCD2	-0.03 (0.70)	*CLDN8*	-0.03 (0.78)
ARHGEF2	-0.03 (0.71)	*CLDN19*	-0.03 (0.78)
CLDN8	0.03 (0.73)	*TRAF4*	-0.02 (0.78)
SHROOM2	0.03 (0.73)	*CLDN12*	-0.02 (0.79)
CGNL1	-0.03 (0.74)	*PARD3B*	0.02 (0.80)
WNK3	0.03 (0.75)	*CDH5*	-0.02 (0.80)
CLDN1	0.03 (0.75)	*VASP*	-0.02 (0.80)
CLDN16	0.03 (0.76)	*CYTH1*	-0.02 (0.81)
LIN7C	-0.03 (0.76)	*ATP7B*	0.02 (0.81)
UBN1	0.03 (0.78)	*OCLN*	0.02 (0.81)
NFASC	-0.02 (0.79)	*SHROOM2*	0.02 (0.83)
NPHP1	-0.02 (0.79)	*RAP2B*	0.02 (0.83)
CLDN7	0.02 (0.8)	*PRKCZ*	0.02 (0.83)

PRKCZ	0.02 (0.82)	*DLG1*	-0.02 (0.83)
POF1B	-0.02 (0.85)	*VAPA*	0.02 (0.84)
CCND1	0.02 (0.85)	*MARVELD3*	-0.02 (0.85)
SIPA1L3	0.02 (0.86)	*GJA1*	0.02 (0.86)
AMOTL2	0.02 (0.86)	*APC*	-0.02 (0.86)
PDCD6IP	0.01 (0.87)	*ANK3*	-0.02 (0.86)
USP53	0.01 (0.89)	*PLXDC1*	0.02 (0.87)
RAP2B	0.01 (0.89)	*RAP2C*	-0.02 (0.87)
PRKCI	0.01 (0.89)	*LIN7C*	-0.02 (0.87)
PMP22	-0.01 (0.91)	*PALS1*	0.02 (0.87)
CRB3	0.01 (0.92)	*EPHA2*	-0.01 (0.88)
PLXDC1	0.01 (0.92)	*TJP1*	-0.01 (0.90)
CDK4	-0.01 (0.92)	*AMOTL2*	0.01 (0.90)
LIN7A	0.01 (0.92)	*MAGI1*	-0.01 (0.90)
MTDH	0.01 (0.92)	*POF1B*	0.01 (0.90)
CTNNB1	0.01 (0.93)	*CGN*	0.01 (0.91)
FRMD4A	0.01 (0.95)	*CTNNB1*	0.01 (0.91)
TJP1	0.00 (0.96)	*FZD5*	-0.01 (0.94)
RAP2C	0.00 (0.98)	*CRB3*	0.00 (0.97)
RPGRIP1L	0.00 (0.98)	*MTDH*	0.00 (0.99)
ADCYAP1R1	0.00 (0.99)	*CLDN17*	0.00 (0.99)
MPDZ	0.00 (0.99)	*CGNL1*	0.00 (1.00)
PARD6G	0.00 (0.99)	*MPDZ*	0.00 (1.00)
Cytokine gene		Cytokine gene	
IL8	0.18 (0.04)	*IL1β*	0.25 (0.01)
VEGF	-0.18 (0.05)	*TGFβ1*	0.18 (0.05)
IL18	0.17 (0.05)	*IL18*	0.14 (0.12)
IL1β	0.13 (0.16)	*SMAD4*	-0.13 (0.14)
SMAD4	-0.10 (0.28)	*IL8*	0.09 (0.33)
TNF	0.09 (0.32)	*VEGF*	-0.08 (0.41)
TGFβ1	0.06 (0.50)	*IL6*	0.07 (0.47)
EGF	0.05 (0.60)	*IL10*	0.05 (0.56)
MMP9	-0.02 (0.85)	*MMP9*	0.05 (0.58)
IL10	0.02 (0.85)	*IL33*	-0.05 (0.60)
IL6	0.01 (0.87)	*IL1α*	0.04 (0.63)
IL1α	0.01 (0.94)	*EGF*	0.02 (0.83)
IL33	0.00 (0.99)	*TNF*	-0.02 (0.85)

[1]Spearman's rank correlation coefficient

P-values shown have not been adjusted for multiple testing. On applying the false discovery rate approach, all adjusted p-values were >0.5.

Supplementary Table 9. Correlations of tight junctions and cytokines gene expression with p24 concentrations measured in foreskin tissue challenged ex vivo with HIV-1$_{BaL}$.

TJ Genes	Correlation[1] with p24 at day 15 following high titre ex vivo challenge (p-value)	TJ Gene	Correlation[1] with p24 at day 15 following low titre ex vivo challenge (p-value)
MICALL2	0.29 (0.12)	*CNTNAP1*	0.28 (0.24)
TJAP1	0.22 (0.82)	*SAPCD2*	0.24 (0.49)
CLDN19	-0.21 (0.76)	*SIPA1L3*	0.22 (0.51)
FRMD4B	0.20 (0.69)	*CLDN15*	0.22 (0.47)
ILDR1	-0.20 (0.67)	*VASP*	0.21 (0.47)
ANK3	0.19 (0.74)	*CYTH2*	0.21 (0.43)
CLDN5	0.19 (0.67)	*LIN7B*	0.20 (0.40)
SYNPO	-0.18 (0.62)	*OCEL1*	0.20 (0.43)
JAML	-0.17 (0.72)	*SH3BP1*	-0.20 (0.39)
RAP2B	0.17 (0.78)	*JAM3*	0.19 (0.47)
ARHGEF2	0.16 (0.78)	*CYTH1*	0.18 (0.48)
WNK3	0.16 (0.79)	*BVES*	0.18 (0.46)
PARD3	0.16 (0.73)	*PARD6A*	0.17 (0.50)
BVES	0.15 (0.84)	*TBCD*	0.17 (0.47)
SHROOM2	-0.15 (0.80)	*RIGI*	0.16 (0.54)
MAPK15	0.15 (0.75)	*SYMPK*	0.16 (0.57)
CLDN7	0.15 (0.73)	*ARHGAP17*	0.16 (0.56)
CNTNAP1	-0.15 (0.69)	*CCDC85C*	0.15 (0.58)
CLDN11	-0.14 (0.69)	*LSR*	0.15 (0.60)
CLDN15	-0.14 (0.68)	*PMP22*	0.15 (0.58)
TJP2	-0.14 (0.75)	*CLDN3*	-0.14 (0.69)
USP53	-0.14 (0.72)	*CLMP*	0.14 (0.68)
RAP2C	-0.13 (0.71)	*NFASC*	0.14 (0.65)
CCND1	0.13 (0.72)	*EPCAM*	-0.14 (0.62)
TRAF4	0.13 (0.72)	*PLXDC1*	0.14 (0.61)
MAGI1	0.13 (0.69)	*POF1B*	-0.14 (0.58)
AOC1	-0.13 (0.67)	*CGN*	0.14 (0.59)
MARVELD2	0.12 (0.73)	*FRMD4B*	0.14 (0.57)
CDH5	0.12 (0.77)	*CLDN5*	0.13 (0.60)
LIN7B	-0.12 (0.75)	*ESAM*	0.13 (0.60)
MPP7	-0.12 (0.74)	*RAB13*	0.13 (0.60)
CGNL1	0.12 (0.74)	*PRKCZ*	-0.13 (0.60)
ADCYAP1R1	0.11 (0.79)	*MXRA8*	-0.12 (0.63)
ARHGAP17	-0.11 (0.79)	*PATJ*	0.12 (0.63)
NPHP4	-0.11 (0.79)	*FZD5*	-0.12 (0.69)
EPHA2	-0.11 (0.80)	*MAPK15*	-0.11 (0.67)
PATJ	0.10 (0.79)	*PARD6B*	0.11 (0.66)
CLDN3	-0.10 (0.90)	*MPDZ*	0.11 (0.65)
MTDH	0.09 (0.90)	*AMOTL2*	0.11 (0.64)
UBN1	-0.09 (0.90)	*CLDN17*	0.11 (0.65)
TJP3	0.09 (0.88)	*CDK4*	0.11 (0.64)
AMOT	0.09 (0.88)	*AMOT*	-0.11 (0.63)
SH3BP1	0.09 (0.88)	*CXADR*	0.11 (0.66)

PRKCZ	0.09 (0.89)	*ASH1L*	0.11 (0.65)
MARVELD3	0.09 (0.87)	*MARVELD3*	0.11 (0.64)
JAM3	0.09 (0.86)	*UBN1*	0.11 (0.63)
ACTB	0.09 (0.87)	*STRN*	0.10 (0.66)
RAPGEF2	0.09 (0.86)	*CLDN10*	0.10 (0.68)
CGN	0.08 (0.86)	*CLDN16*	-0.10 (0.68)
OCEL1	-0.08 (0.84)	*CRB3*	0.09 (0.70)
AMOTL2	-0.08 (0.84)	*ARHGEF2*	0.09 (0.78)
OCLN	0.08 (0.83)	*AOC1*	0.08 (0.84)
CYTH3	-0.08 (0.85)	*CLDN23*	-0.08 (0.82)
PARD6G	0.08 (0.83)	*CLDN1*	-0.08 (0.81)
ASH1L	0.08 (0.82)	*AFDN*	0.08 (0.84)
MAGI3	-0.08 (0.85)	*GJA1*	0.08 (0.84)
LIN7C	-0.08 (0.84)	*ILDR1*	-0.07 (0.85)
PARD6A	-0.07 (0.86)	*JAM2*	0.07 (0.85)
MAGI2	0.07 (0.87)	*CLDN8*	-0.07 (0.83)
SYMPK	-0.07 (0.87)	*CTNNB1*	0.07 (0.87)
CLDN23	0.07 (0.87)	*MAGI2*	0.07 (0.88)
AMOTL1	0.07 (0.89)	*WNK3*	0.07 (0.87)
EPPK1	0.07 (0.88)	*MARVELD2*	-0.07 (0.87)
CLDN4	0.06 (0.89)	*TRAF4*	-0.06 (0.89)
MXRA8	-0.06 (0.89)	*JAML*	0.06 (0.88)
RIGI	0.06 (0.90)	*PALS1*	-0.06 (0.89)
NPHP1	0.06 (0.90)	*ACTB*	0.06 (0.89)
CLDN10	0.06 (0.89)	*CLDN7*	0.06 (0.90)
RAB13	0.06 (0.89)	*ATP7B*	0.06 (0.91)
RPGRIP1L	0.06 (0.88)	*LIN7C*	0.06 (0.91)
APC	0.06 (0.88)	*CLDN11*	0.06 (0.91)
PLXDC1	0.06 (0.87)	*EPPK1*	-0.05 (0.90)
CLDN16	-0.06 (0.87)	*TGFBR1*	0.05 (0.90)
CXADR	-0.06 (0.87)	*TJP1*	0.05 (0.90)
PMP22	0.05 (0.92)	*TJAP1*	-0.05 (0.91)
LIN7A	-0.05 (0.91)	*CYTH3*	0.05 (0.94)
FRMD4A	0.05 (0.93)	*ECT2*	-0.05 (0.93)
CLDN8	-0.05 (0.93)	*CCND1*	0.05 (0.93)
EPB41L4B	0.05 (0.92)	*WNK4*	-0.05 (0.92)
DLG3	-0.04 (0.93)	*PARD3*	-0.04 (0.94)
POF1B	-0.04 (0.92)	*TJP3*	-0.04 (0.95)
F11R	0.04 (0.92)	*TJP2*	-0.04 (0.94)
PARD6B	-0.04 (0.91)	*RPGRIP1L*	-0.04 (0.94)
FZD5	0.04 (0.91)	*CDH5*	-0.04 (0.94)
CLDN12	-0.04 (0.91)	*SYNPO*	0.04 (0.93)
DLG1	0.04 (0.90)	*CLDN12*	-0.04 (0.92)
LSR	-0.04 (0.90)	*CLDN4*	-0.04 (0.91)
PRKCI	-0.04 (0.93)	*CGNL1*	-0.04 (0.92)
PDCD6IP	0.03 (0.95)	*NPHP1*	-0.03 (0.98)
STRN	-0.03 (0.95)	*PRKCI*	-0.03 (0.97)
GJA1	0.03 (0.97)	*EPB41L4B*	0.03 (1.00)
JAM2	0.03 (0.97)	*PDCD6IP*	0.02 (1.00)
CDK4	-0.03 (0.98)	*PARD3B*	-0.02 (1.00)

TBCD	-0.03 (0.98)	*SHROOM2*	0.02 (0.99)
PALS1	0.02 (0.98)	*AMOTL1*	-0.02 (1.00)
CLDN1	-0.02 (0.98)	*APC*	0.02 (1.00)
MPDZ	0.02 (0.97)	*RAP2B*	-0.02 (0.99)
CYTH1	-0.02 (0.99)	*PARD6G*	0.02 (0.98)
CRB3	-0.02 (1.00)	*MICALL2*	-0.02 (1.00)
AFDN	0.02 (1.00)	*MPP7*	0.01 (1.00)
WNK4	0.02 (1.00)	*MTDH*	0.01 (1.00)
ECT2	-0.02 (1.00)	*DLG3*	-0.01 (1.00)
ATP7B	0.01 (1.00)	*FRMD4A*	-0.01 (1.00)
SAPCD2	-0.01 (1.00)	*USP53*	0.01 (1.00)
CLMP	0.01 (1.00)	*ANK3*	-0.01 (1.00)
CCDC85C	0.01 (1.00)	*EPHA2*	0.01 (1.00)
EPCAM	-0.01 (1.00)	*F11R*	0.01 (1.00)
NFASC	-0.01 (1.00)	*DLG1*	0.01 (1.00)
SIPA1L3	0.01 (1.00)	*MAGI1*	-0.01 (1.00)
VASP	-0.01 (0.99)	*MAGI3*	-0.01 (1.00)
CTNNB1	0.01 (0.99)	*LIN7A*	-0.01 (1.00)
CYTH2	-0.01 (0.98)	*ADCYAP1R1*	-0.01 (1.00)
TGFBR1	0.00 (1.00)	*NPHP4*	-0.01 (1.00)
PARD3B	0.00 (1.00)	*CLDN19*	0.00 (1.00)
ESAM	0.00 (1.00)	*RAPGEF2*	0.00 (1.00)
VAPA	0.00 (1.00)	*RAP2C*	0.00 (1.00)
TJP1	0.00 (0.99)	*OCLN*	0.00 (1.00)
CLDN17	0.00 (1.00)	*VAPA*	0.00 (1.00)

[1]Spearman's rank correlation coefficient

P-values shown have been adjusted for multiple testing using the false discovery rate approach.

Supplementary Table 10. Effect of oral PrEP on expression of tight junction proteins detected by Western blot.

Protein	PrEP = None	PrEP = FTC-TDF	PrEP = FTC-TAF	p-value
**All participants**				
Claudin-1	0.31 (0.04, 0.74)	0.27 (0.13, 0.69)	0.28 (0.14, 0.74)	0.91
Occludin	2.46 (0.54, 5.58)	2.25 (0.59, 4.81)	1.55 (0.77, 4.03)	0.93
Zonula occludens-1	0.008 (0.001, 0.023)	0.01 (0.003, 0.05)	0.01 (0.0002, 0.05)	0.65

Claudin-1 coded by *CLDN-1*; Occludin coded by *OCLN*; Zonula occludens-1 (ZO-1) coded by *TJP1* gene.

Median expression (with interquartile range in parentheses) by trial arm, and p-value for differences between trial arms from Kruskal-Wallis test are shown.

Supplementary Figure 1. **Heatmap showing expression levels (gene counts) of 118 TJ genes for 139 individuals included in the CHAPS study.** Each row represents one individual, and each column represents one gene. Individuals (rows) are grouped by country, and ordered in the chronological order in which they were enrolled in the study. Genes (columns) are ordered by median gene expression level (high to low). Darker red values indicate higher gene counts while darker blue values indicate low gene counts, as shown in the legend.

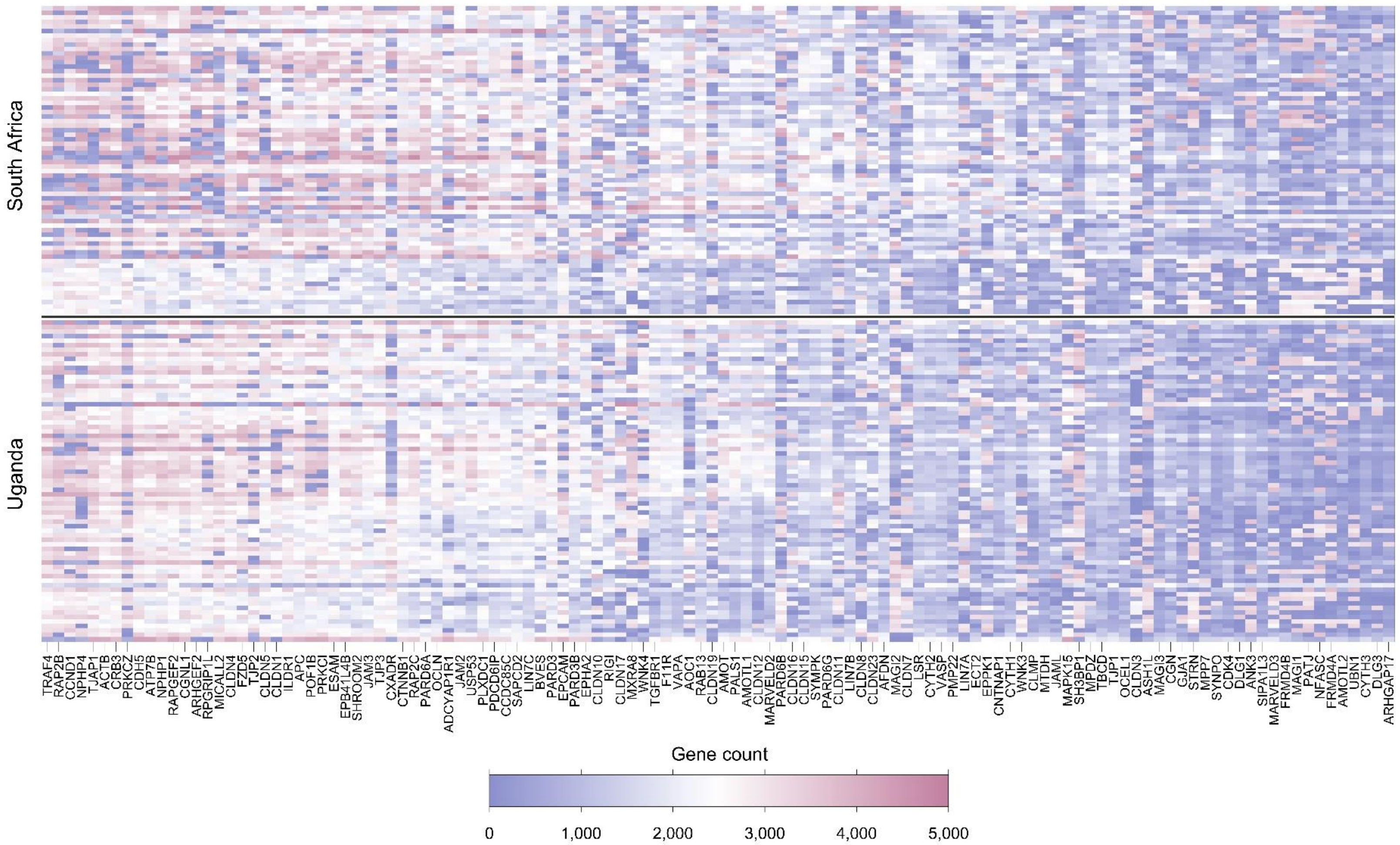

Supplementary Figure 2. Heat map showing pairwise correlation coefficients between tight junction gene expression levels. Each row and column represent one of the 118 TJ genes Spearman's correlation coefficient was calculated between expression levels for each pairwise combination of TJ genes. Darker red values indicate higher positive correlation while darker blue values indicate higher negative correlation, as shown in the legend.

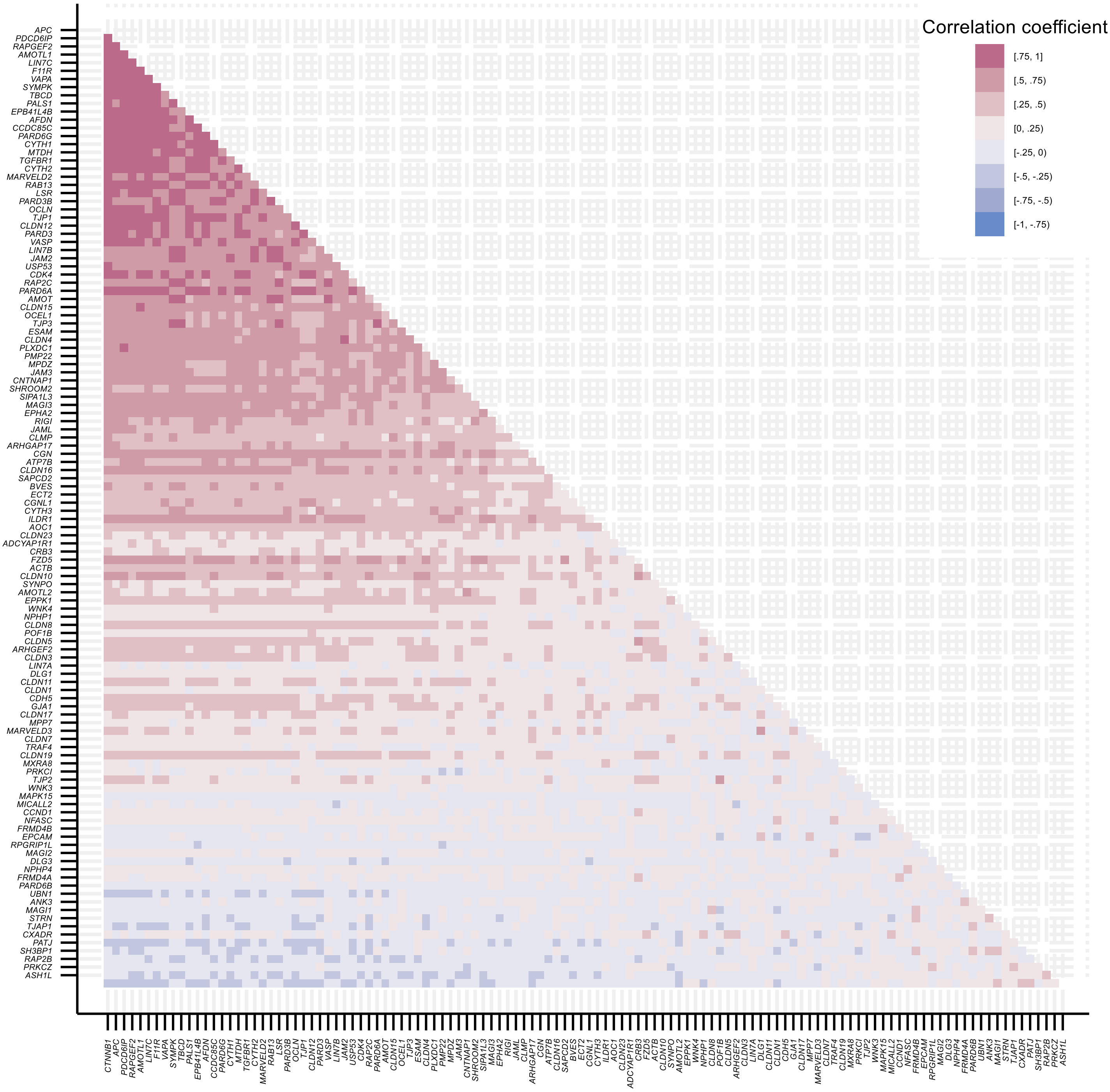

Supplementary Figure 3. Distribution of scores for first principal component, capturing 31% of the variability in expression levels of 118 genes, by trial arm. Principal components analysis was used to generate a new variable (the first principal component; component 1) comprising a linear combination of the 118 TJ gene expression levels and capturing 31% of their variability; a high score for this component represents high expression levels for many of the TJ genes, while a low score for this component represents low expression levels for many of the TJ genes. Boxplots show the distribution of scores for the first principal component, with values indicated on the y-axis. Distributions are shown separately by the type of PrEP received by participants, as indicated on the x-axis.

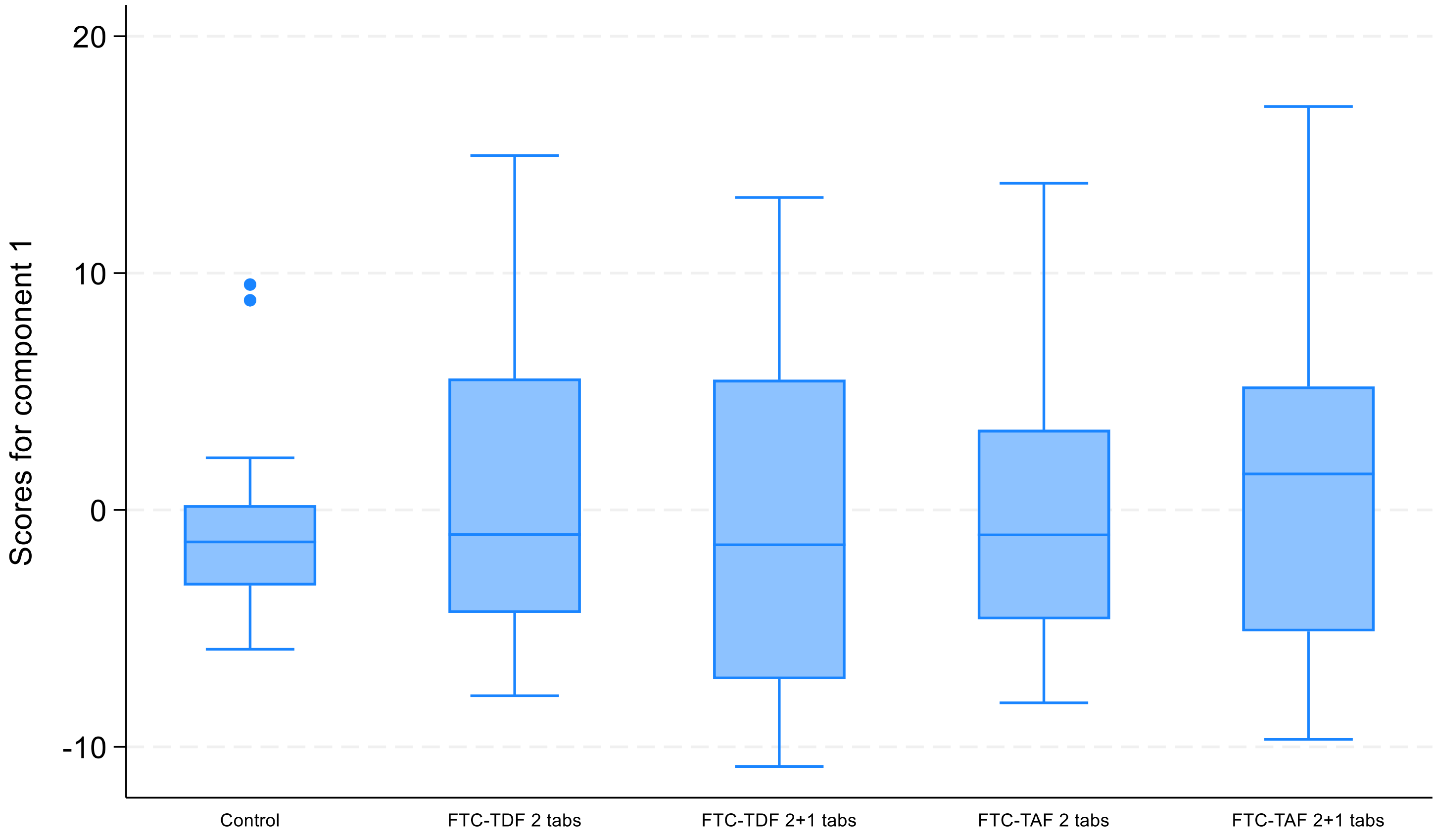

Supplementary Figure 4. Correlation between scores from first principal component (representing 31% of the variability in the 118 genes) and cytokine gene counts. Principal components analysis was used to generate a new variable (the first principal component; component 1) comprising a linear combination of the 118 TJ gene expression levels and capturing 31% of their variability; a high score for this component represents high expression levels for many of the TJ genes, while a low score for this component represents low expression levels for many of the TJ genes. Each graph shows a scatterplot of the first component score (y-axis) plotted against the cytokine gene counts (x-axis). On each graph, participants from South Africa are shown in grey while participants from Uganda are shown in red. Spearman's correlation coefficients for the correlation between the first component score and the gene count were calculated separately for each country and are indicated on each graph.

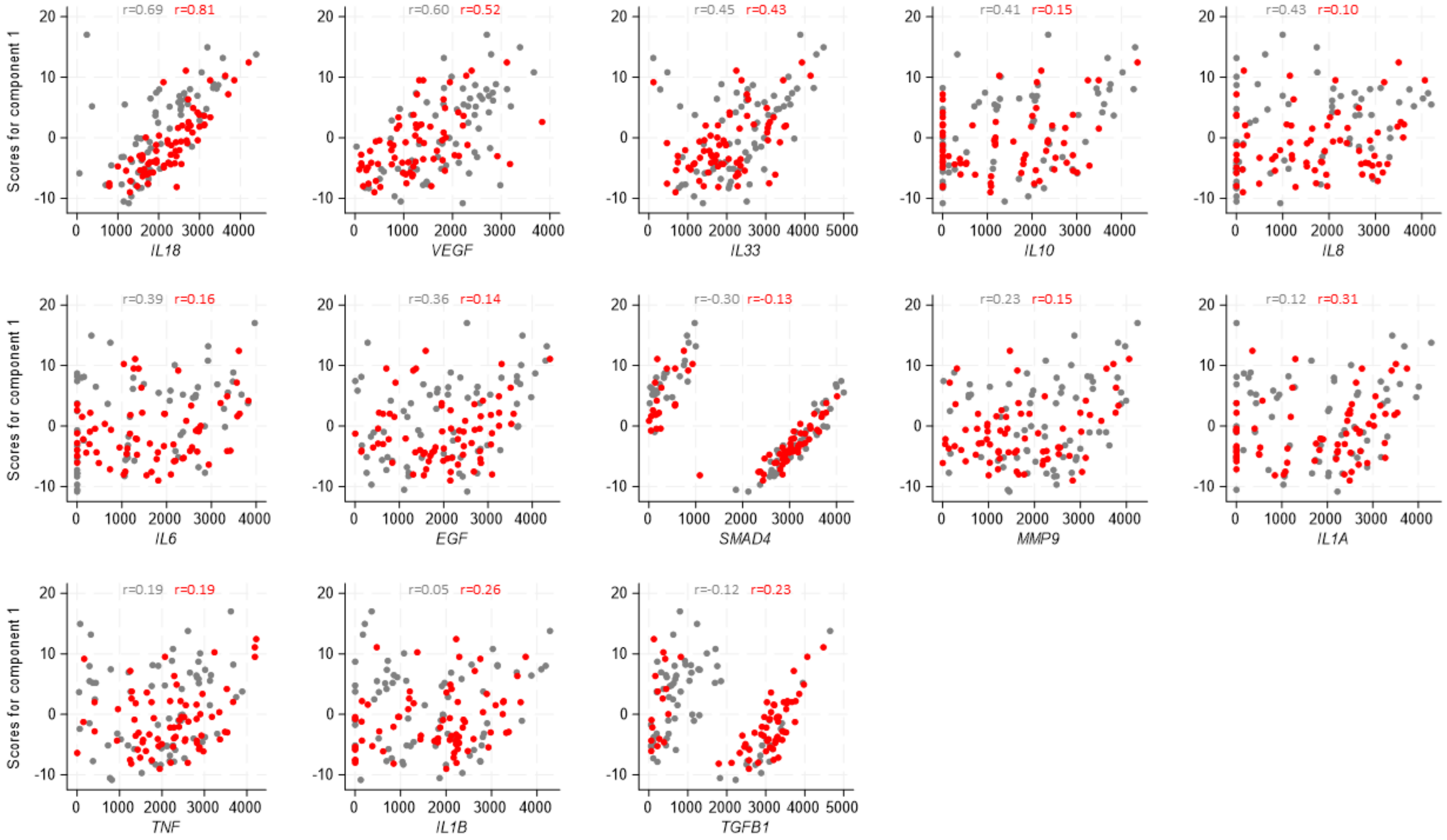

Supplementary Figure 5. Representative western blot for tight junction levels in foreskin tissue. Nitrocellulose membranes were cut in four sections and blotted with anti-ZO-1, anti-OCLN, anti-CLDN-1 or anti-[illegible]-actin antibodies. Image shown is from 13 samples loaded on one gel by date of enrolment.

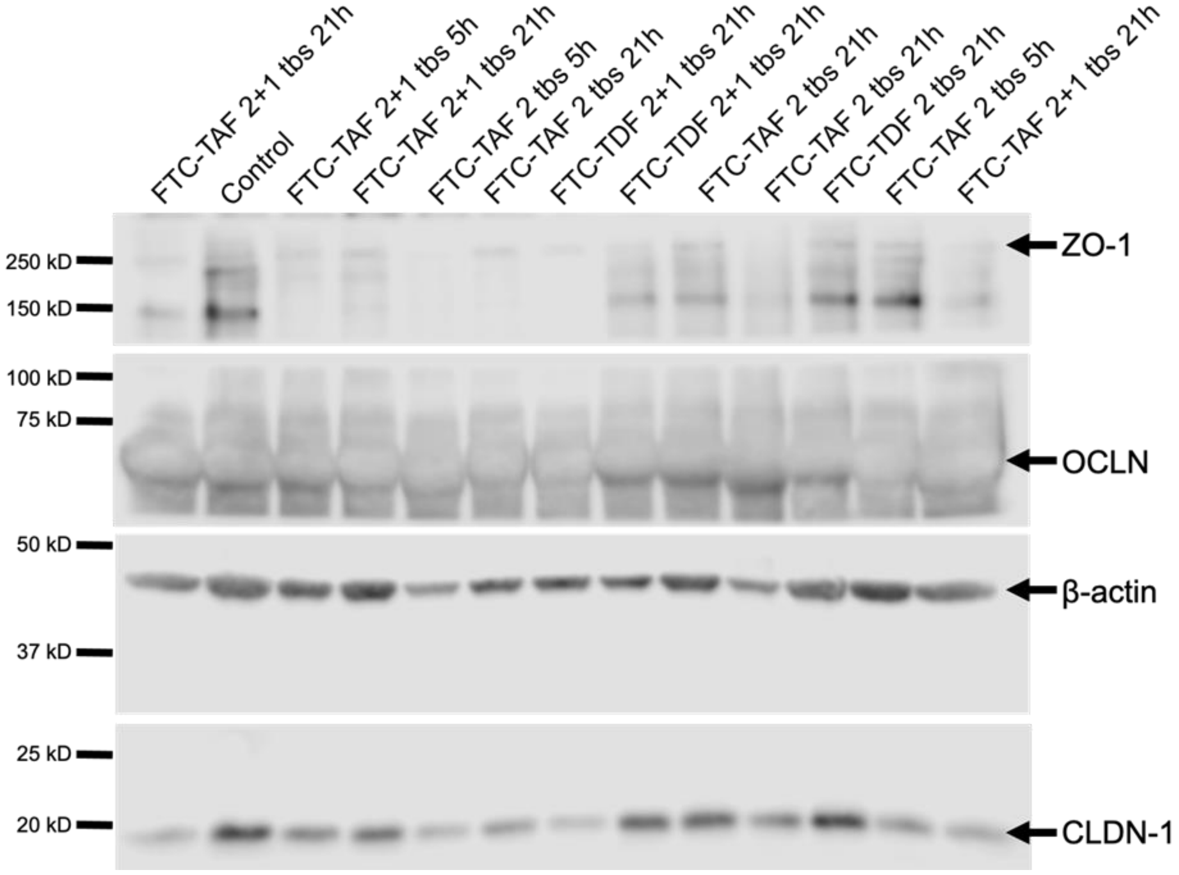

Supplement: Supplementary file 1 [file DataSheet1.pdf]
